# Chemical reactions for the ligand-directed modification of native proteins

**DOI:** 10.1039/d6cs00702c

**Published:** 2026-10-08

**Authors:** Anissa Haim, Cassandra J. Henderson, Merve Akdeniz, Timothy Noël, Tom N. Grossmann

**Affiliations:** a Department of Chemistry and Pharmaceutical Sciences, VU University Amsterdam De Boelelaan 1108 1081 HZ Amsterdam The Netherlands; b Flow Chemistry Group, Van’t Hoff Institute for Molecular Sciences (HIMS), University of Amsterdam Science Park 904 1098 XH Amsterdam The Netherlands t.noel@uva.nl; c Institute of Organic and Biomolecular Chemistry, Georg-August-Universität Göttingen Tammannstr. 2 37077 Göttingen Germany tom.grossmann@uni-goettingen.de

## Abstract

Selective modifications of native proteins provide powerful opportunities to study and manipulate biological systems, yet achieving site-selective functionalization remains challenging due to the surfeit of competing amino acids, often addressed by genetic engineering of target proteins. Ligand-directed protein modification addresses these limitations by exploiting reversible molecular recognition to position reactive groups in proximity to a defined protein site, thereby enabling selective covalent modification of native proteins under physiological conditions. Importantly, ligand-directed protein modification is evolving from a specialized labeling methodology into a versatile platform for the selective manipulation of protein function in native biological environments. In this review, we discuss recent advances in ligand-directed strategies that employ synthetically accessible small molecules and peptides as recognition elements. Current approaches are categorized into (i) ligation reactions, in which the ligand remains attached to the protein, (ii) transfer reactions, which install a functional moiety while releasing the ligand, and (iii) ligand-directed catalysis, where ligand-tethered catalysts promote protein-substrate reactions with catalytic turnover. For each strategy, employed chemical reactions, amino acid selectivities, and applications in chemical biology and biotechnology are described. We further compare the strengths and limitations of these approaches considering the balance between reactivity, selectivity, and biocompatibility.

## Abstract 2

This tutorial review introduces the principles of ligand-directed protein modification and compares the chemical strategies that enable selective covalent functionalization of native proteins through ligation, transfer, and catalysis.

## Graphical abstract

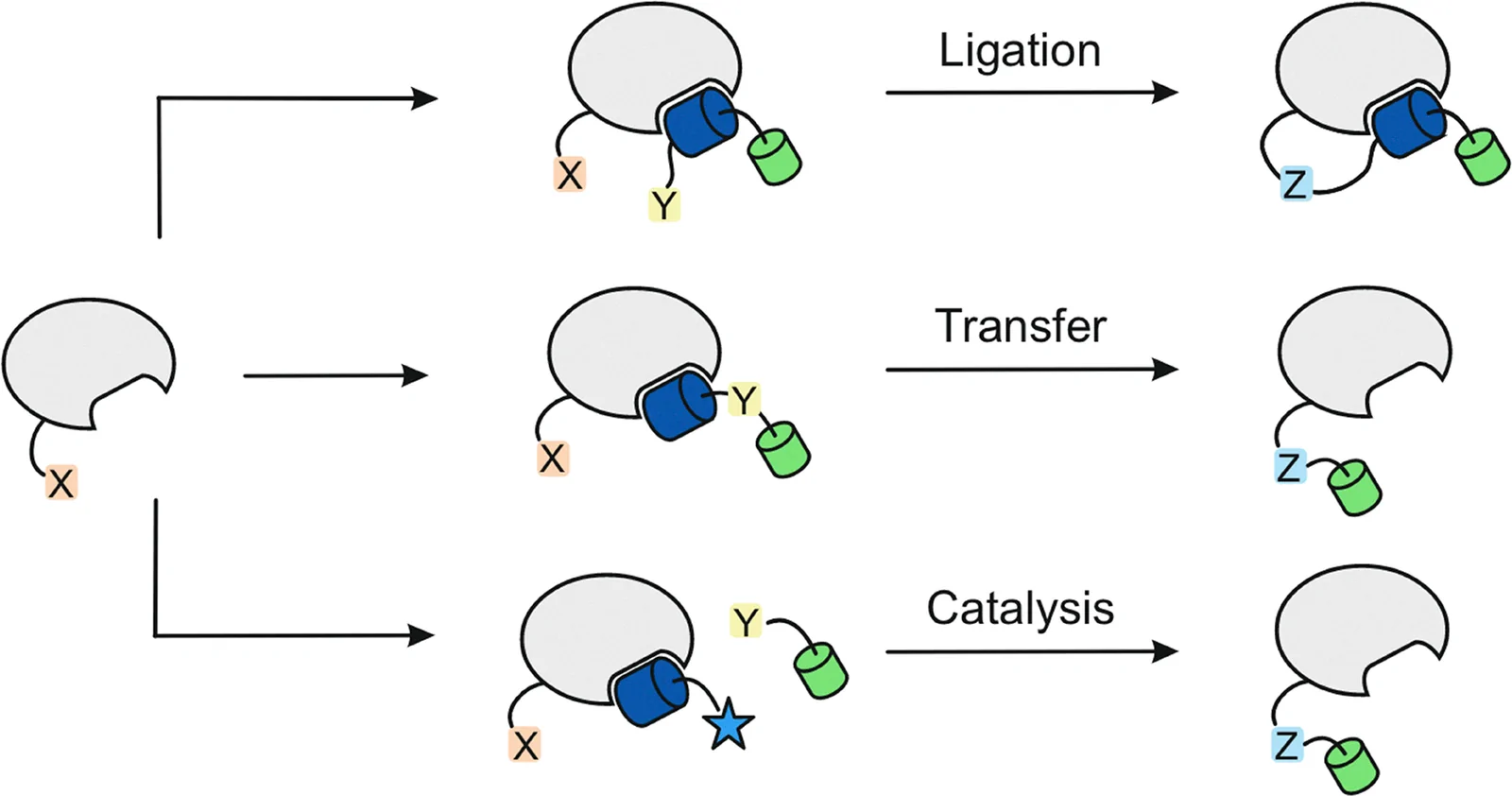

**Key learning points**

1. The principles of ligand-directed protein modification for the selective functionalization of native proteins.

2. The mechanistic distinctions between ligation, transfer, and proximity-guided catalytic protein modification strategies.

3. The factors that govern efficient and site-selective ligand-directed protein modification.

4. The strengths, limitations, and design trade-offs of current ligand-directed protein modification approaches.

5. Emerging strategies to expand the scope, precision, and biological applicability of ligand-directed protein modification.

## 1. Introduction

The selective chemical modification of proteins under native conditions is an efficient strategy to study and manipulate biological systems.^1–3^ The ability to install new functionalities onto proteins is essential for various research fields ranging from chemical biology to the development of therapeutics and biomaterials. However, achieving precise control over the site of modification remains challenging, as protein surfaces typically present multiple amino acid residues with similar reactivity. Traditional bioconjugation approaches often rely on the intrinsic reactivity of amino acid residues resulting in a limited chemo- and regioselectivity.^4,5^ This limitation is particularly relevant to antibody-drug conjugates (ADC), where conventional lysine acylation or cysteine alkylation can produce heterogeneous conjugate mixtures, complicating the precise control of drug loading and conjugation sites that is desirable for drug development and manufacturing.^6^ Alternative methods include the incorporation of genetic fusion tags, specific peptide motifs, or non-canonical amino acids to achieve site-selectivity, however, their reliance on genetic manipulation of the target protein is incompatible with applications that require the use of the native protein sequence.^7–10^

The exploitation of proximity effects to control chemical reactivity is a common strategy in biological systems. Rather than relying on high reactant concentrations, nature achieves efficient and selective transformations by positioning reaction partners in close proximity through molecular recognition. Such spatial preorganization increases the effective molarity of reactants, lowers entropic penalties, and enables chemical reactions with exceptional rates and selectivity. Following this principle, ligand-directed chemistry has gained increasing attention as a tool for selective protein modification, harnessing reversible, non-covalent recognition events to position reactive functionalities in close proximity to a defined protein site. This proximity-driven reactivity then enables the selective modification of native proteins without the need for genetic engineering, thereby expanding the scope of chemical biology to systems that are otherwise difficult to access. A central appeal of ligand-directed chemistry lies in its ability to install chemical functionality on proteins without perturbing their primary structure, introducing exogenous tags, or relying on enzymatic catalytic residues.^2,3^ Because selectivity is governed by the binding event and not only relies on inherent residue reactivity, this approach can modify proteins lacking classical reactive “hotspot” nucleophiles or residues that are otherwise difficult to target.

Over the past two decades, this concept has evolved into a versatile toolkit capable of labeling purified proteins as well as target proteins in live cells,^2,11^ enabling diverse applications, including activity profiling, imaging, target validation, proximity mapping, and the construction of synthetic biosensors.^12–16^ The design requirements can differ substantially: modification of an isolated protein generally tolerates modest selectivity, whereas modification *in vivo* requires highly efficient target recognition and minimal off-target reactivity to selectively modify the target protein in the presence of an excess of other cellular proteins. Overall, three main classes of ligand-directed protein modification reactions can be distinguished (Fig. 1): (i) ligation reactions in which the ligand remains part of the final protein conjugate, (ii) transfer reactions which involve the release of the ligand, allowing protein modifications that comprise only the functional moiety without introducing the steric footprint of the ligand scaffold, and (iii) ligand-directed catalysis employing ligand-tethered catalysts to promote reactions on the protein surface, enabling turnover and the use of reagent classes that cannot be directly appended to ligands.

**Fig. 1:**
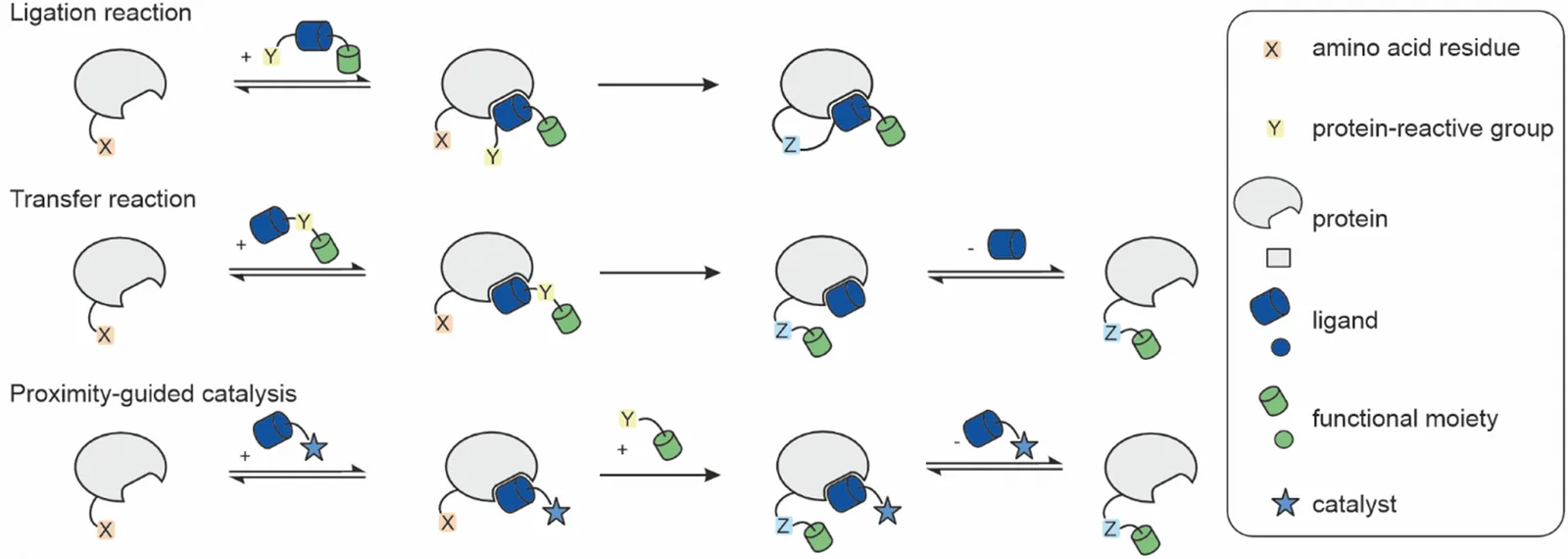
Overview of ligand-directed protein modification: ligation reactions (top), in which the ligand forms a covalent bond with a target amino acid residue. Transfer reactions (middle), in which the ligand is released after the modification. Proximity-guided catalysis (bottom), where a ligand-bound catalyst promotes a chemical reaction successively on multiple target protein molecules.

This review summarizes recent advances across the three major categories, focusing on the use of ligands with a relatively small molecular weight including natural products, small molecules and peptides which can be obtained synthetically. Ligands comprising proteins as well as oligonucleotides are not included. Also, covalent inhibitors that target catalytic residues in the active site of enzymes are not considered and have been summarized elsewhere.^17,18^ Herein, we focus on strategies that allow the chemical modification of native proteins (*i.e.*, untagged and genetically unmodified) highlighting design strategies, the chemical reactions employed for protein modification, and their residue selectivity, and finally discussing the opportunities and current limitations of ligand-directed protein modification.

## 2. Ligation reactions

Ligand-directed protein modification through chemical ligation recapitulates the most direct form of proximity-driven covalent labeling. In these approaches, a synthetic ligand binds the target protein and positions a reactive group near a suitable residue. Upon bond formation, the ligand remains attached to the protein.^17,19^ The efficiency and specificity of ligation reactions are governed by the orientation, affinity and binding kinetics of the ligand-protein complex, and the intrinsic reactivity of the reactive moiety as well as the target residue. In the following section, ligand-directed ligation reactions are outlined distinguishing between permanent conjugation (Fig. 2, top) and ligations that are followed by a cleavage reaction to release the ligand and introduce the functional moiety (Fig. 2, bottom).

**Fig. 2:**
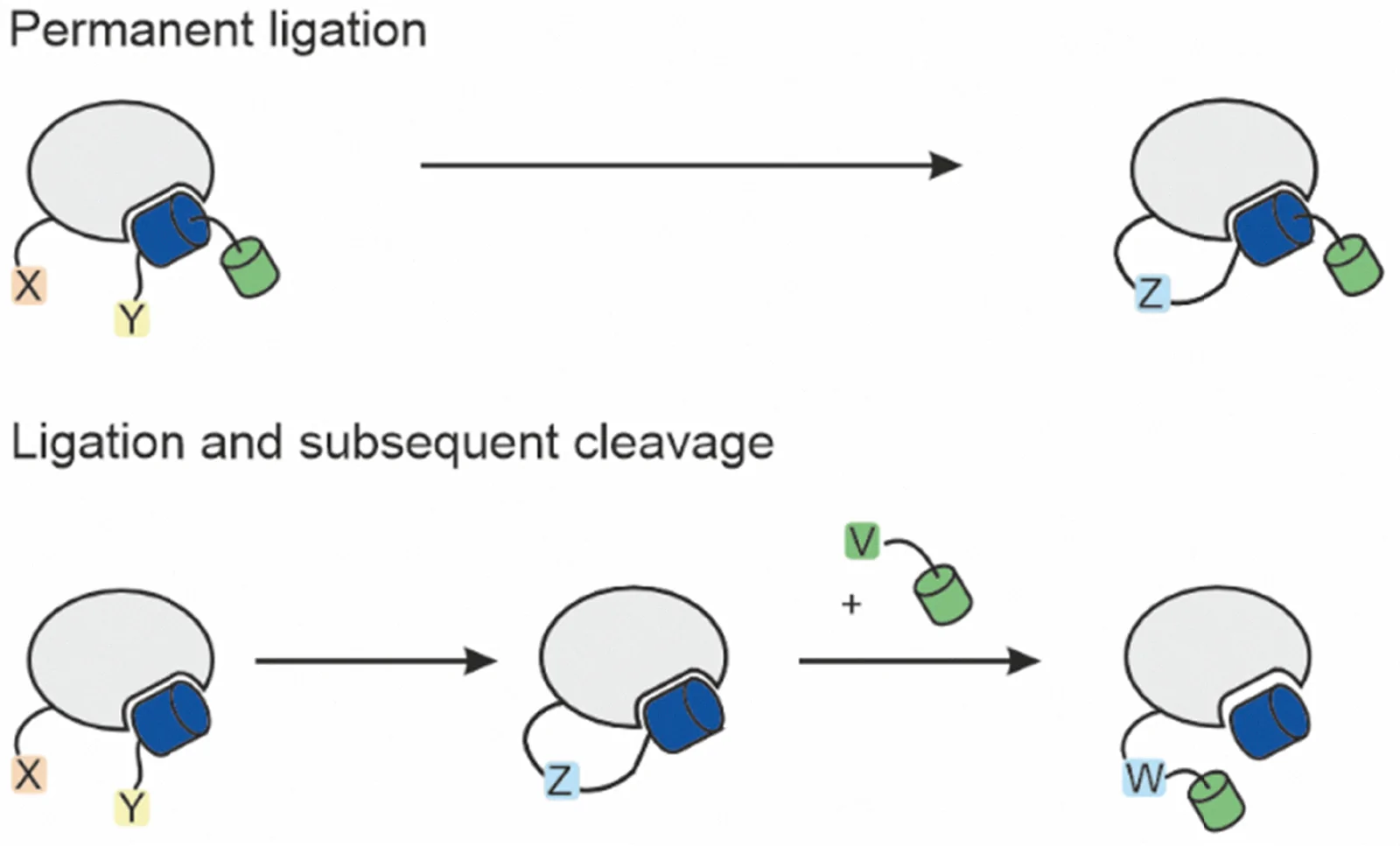
Overview of the two ligation strategies involving permanent ligations (top) and ligation reactions with subsequent cleavage to release the ligand (bottom).

### 2.1 Permanent ligation

A ligation reaction generates a covalent bond linking ligand and target protein. The underlying reaction type defines which amino acid residues can be addressed, the stability of the linkage, and the steric consequences of the modification. In most ligand-directed systems, selectivity arises from a balance between the inherent reactivity of the electrophile and the proximity effect achieved *via* ligand binding. Consequently, electrophiles with a wide range of reactivities can be employed, provided that ligand binding sufficiently promotes the reaction with the desired target residue. This distinguishes them from techniques aimed primarily at target identification or interaction mapping, such as activity-based protein profiling and related covalent capture approaches, which are generally designed to identify or capture protein targets rather than to achieve defined, site-selective installation of functional labels on native proteins.^20,21^

#### 2.1.1 Photoaffinity labeling

Photoaffinity labeling is a historically important approach related to ligand-directed ligation, in which light-activated functional groups generate highly reactive intermediates that covalently capture nearby residues.^22–25^ While this strategy relies on ligand binding to position the reactive group in vicinity of the target protein, covalent bond formation does not proceed spontaneously upon formation of the ligand-protein complex but is only triggered upon irradiation. The formed highly-reactive species can then typically react non-specifically with a variety of residues in proximity resulting in a mixture of products, rather than modification of a single, predictable site. Photoaffinity labeling is primarily employed for target identification, binding-site mapping, and the capture of transient protein-ligand interactions.

Three classes of photoreactive groups are most commonly used. Aryl azides (1) undergo photolysis to generate nitrenes^26,27^ (2, Fig. 3a), diazirines (4) yield highly reactive carbenes^28,29^ (5, Fig. 3b) and benzophenones^30,31^ (7) form triplet diradicals (8) capable of hydrogen-atom abstraction (9, Fig. 3c). Although residue selectivity is governed primarily by binding-site geometry rather than intrinsic side-chain reactivity, each photoreactive group reacts preferentially, though not exclusively, with certain groups of residues.^32^ Among these, diazirines have become the preferred photoreactive motif due to their small steric footprint, fast crosslinking kinetics, and activation at relatively long UV wavelengths, which minimizes perturbation of ligand binding and reduces photodamage to biological samples.^33^

**Fig. 3:**
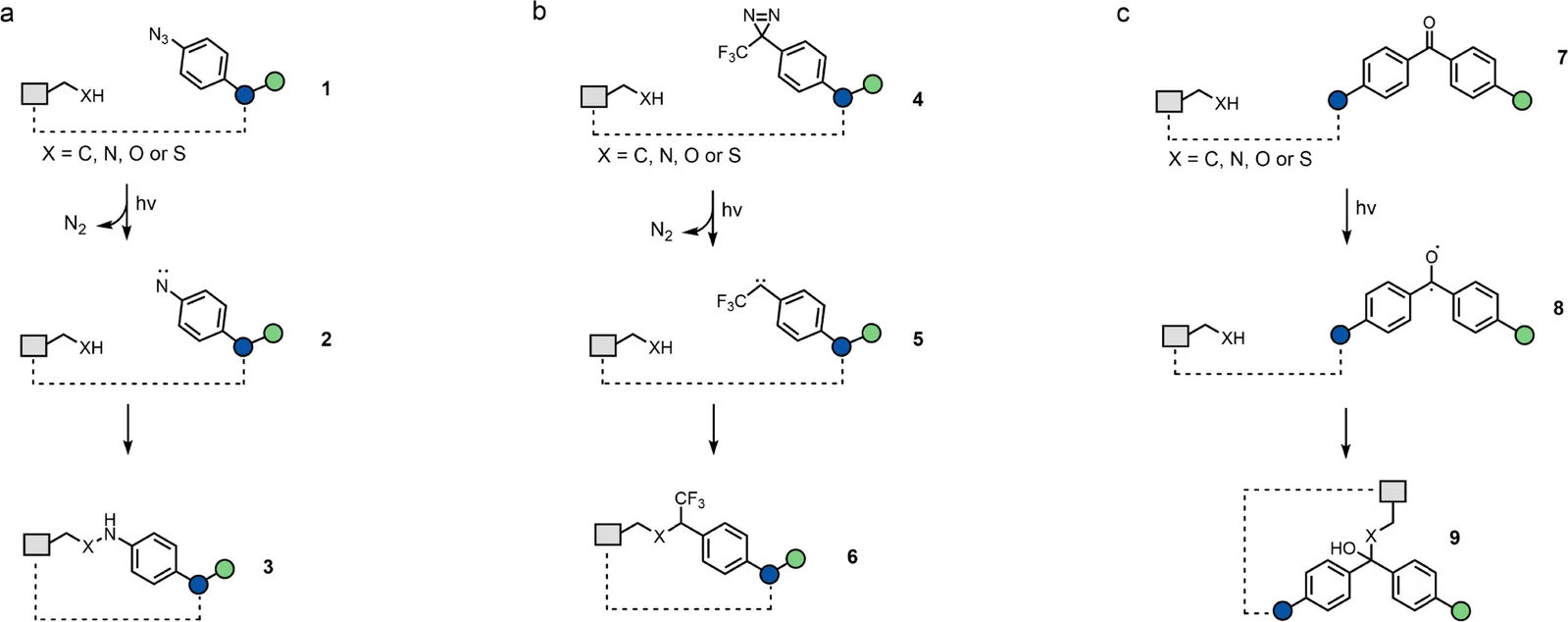
Photoaffinity labeling with (a) aryl azides (1) forming nitrenes (2), (b) diazirines (4) forming carbenes (5) and (c) benzophenones (7) forming diradicals (8).

#### 2.1.2 Addition reactions

Addition reactions provide a versatile platform for ligand-directed protein modification. Once a ligand engages its target, nucleophilic residues can add directly to electrophilic π-systems engineered into the ligand, forming stable C–S or C–N bonds under mild conditions. Additions have been widely exploited to achieve selective covalent capture of native proteins. Acrylamides are commonly used for cysteine-labeling and have also been used in proximity-induced reactions.^34–39^ A representative example is the development of fluorescent peptide probes for monitoring bromodomain activity in cells.^40^ In this system, a peptide ligand was equipped with an acrylamide (10) for a cysteine-selective Michael addition and a nitrobenzoxadiazole fluorophore for signal generation. Upon binding to the bromodomain, a proximal cysteine residue attacks the acrylamide, generating a covalently linked fluorescent peptide–protein conjugate (11, Fig. 4a). Fluorescence intensity increased over time, confirming successful ligation, while incubation with other non-binding proteins showed no obvious increases in fluorescence. More recently, Waser and colleagues introduced a ligand-directed cysteine bioconjugation method that uses peptides equipped with hypervalent iodine electrophiles (12, EBX reagents) to promote thiol addition under aqueous conditions (Fig. 4b).^41^ Both alkyl- and aryl-EBX conjugates yielded stable S-VBX adducts (13) in which the hypervalent iodine motif remains associated with the newly formed C–S bond, effectively generating a peptide–protein conjugate with conversions ranging from 60–90%, depending on the EBX substituent. These S-VBX products were compatible with downstream functionalization such as strain-promoted azide–alkyne cycloaddition (SPAAC) on installed handles, and could be chemically manipulated in a subsequent step, including reductive conditions that removed the hypervalent iodine linkage or, under specific buffer/reductant conditions, even reverted to the native protein.

**Fig. 4:**
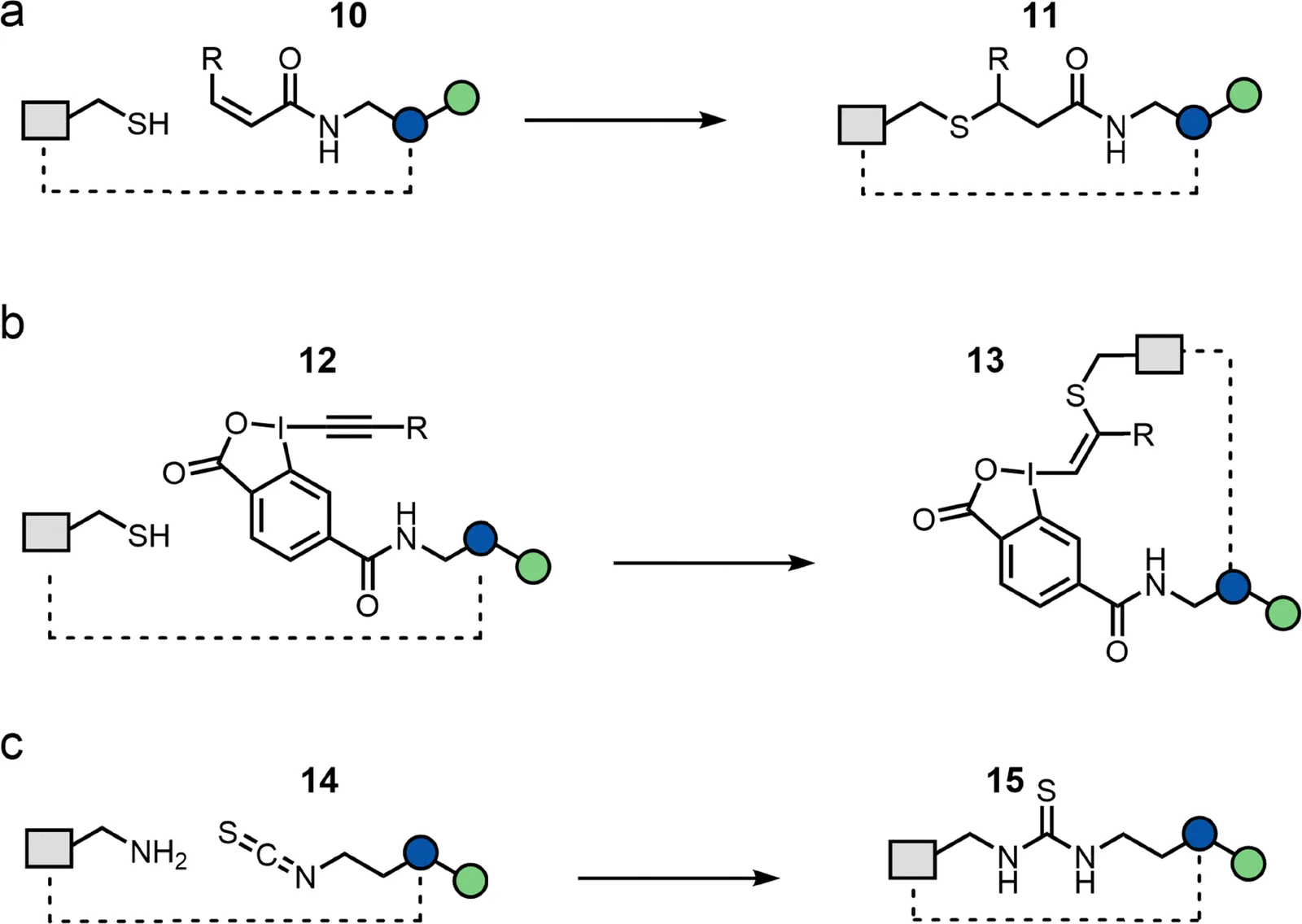
Addition reactions with (a) an acrylamide (10, R = H or substituted), (b) hypervalent iodine electrophiles (12, R = alkyl or aryl group), and (c) an isothiocyanate (14).

Addition reactions can also be directed toward the primary amine in a solvent-exposed lysine residue.^42^ Zhang and coworkers demonstrated that low-to-moderately reactive electrophiles, such as isothiocyanates, phenyl esters, or fluorodinitrobenzene derivatives, could be installed on peptide ligands to achieve site-selective lysine modification in proteins containing multiple lysines (Fig. 4c).^43^ In the case of isothiocyanate electrophiles (14), ligand binding positions the reactive group in close proximity to a lysine residue, enabling formation of a stable thiourea linkage (15) while suppressing nonspecific labeling. This strategy proved effective across a range of proteins, including Src homology 3 (SH3) domains, SUMO proteins as well as ATG8/LC3 family members, and retained selectivity in complex cell lysates.^43^ Additionally, an isocyanate electrophile can be generated *in situ* by sulfonyl fluoride-induced cleavage of a hydroxamic acid-pyrrolidine precursor.^44^

Beyond their established role in protein labeling, acrylamides have also been the dominant electrophile in covalent inhibitors targeting non-catalytic residues, such as in KRAS G12C inhibitors^45,46^ and the first-generation Bruton's tyrosine kinase (BTK) inhibitor ibrutinib.^47,48^ However, their relatively high reactivity can result in the modification of other proteins bearing accessible cysteines^49^ motivating the development and use of milder electrophiles. For example, a ligand bearing a 2-butynamide warhead has been employed to target BTK, exploiting its slower reaction kinetics relative to acrylamide to improve selectivity over related kinases.^50^

#### 2.1.3 Nucleophilic substitutions

Nucleophilic substitution in ligand-directed ligation relies on installing a well-positioned leaving group on the ligand, enabling displacement by a nearby nucleophilic residue once the complex forms. The nature of the leaving group and the geometry induced by ligand binding strongly influence reaction rate and residue selectivity. In this context a broad range of electrophiles, including α-halo carbonyls, activated aryl fluorides, sulfonyl fluorides, *N*-hydroxysuccinimide (NHS) esters, and strained epoxides, have been established in protein labeling^51–53^ and also applied for the selective modification of native proteins.

An early demonstration of this concept was provided by the Meares group, who incorporated chloroacetamide electrophiles (16) in peptide ligands to achieve a proximity-dependent reaction with a lysine (Fig. 5a).^54^ A 19-mer peptide bearing a dinitrofluorobenzene group showed enhanced target engagement with VEGF, resulting from efficient, proximity-driven displacement of the fluoride leaving group upon binding (19). A complementary approach was later developed by Lu and coworkers, who engineered protein–peptide pairs to undergo affinity-guided and site-specific cysteine alkylation *via* α-chloroacetyl substitution (16, Fig. 5a).^55^ Each protein contained a strategically positioned cysteine residue that reacted with a chloroacetyl electrophile built into its cognate peptide ligand, forming an irreversible thioether through an SN2 reaction. These reactions were fast, selective, and operational under physiological conditions, enabling applications such as receptor labeling and assembly of multi-protein complexes in purified systems.

**Fig. 5:**
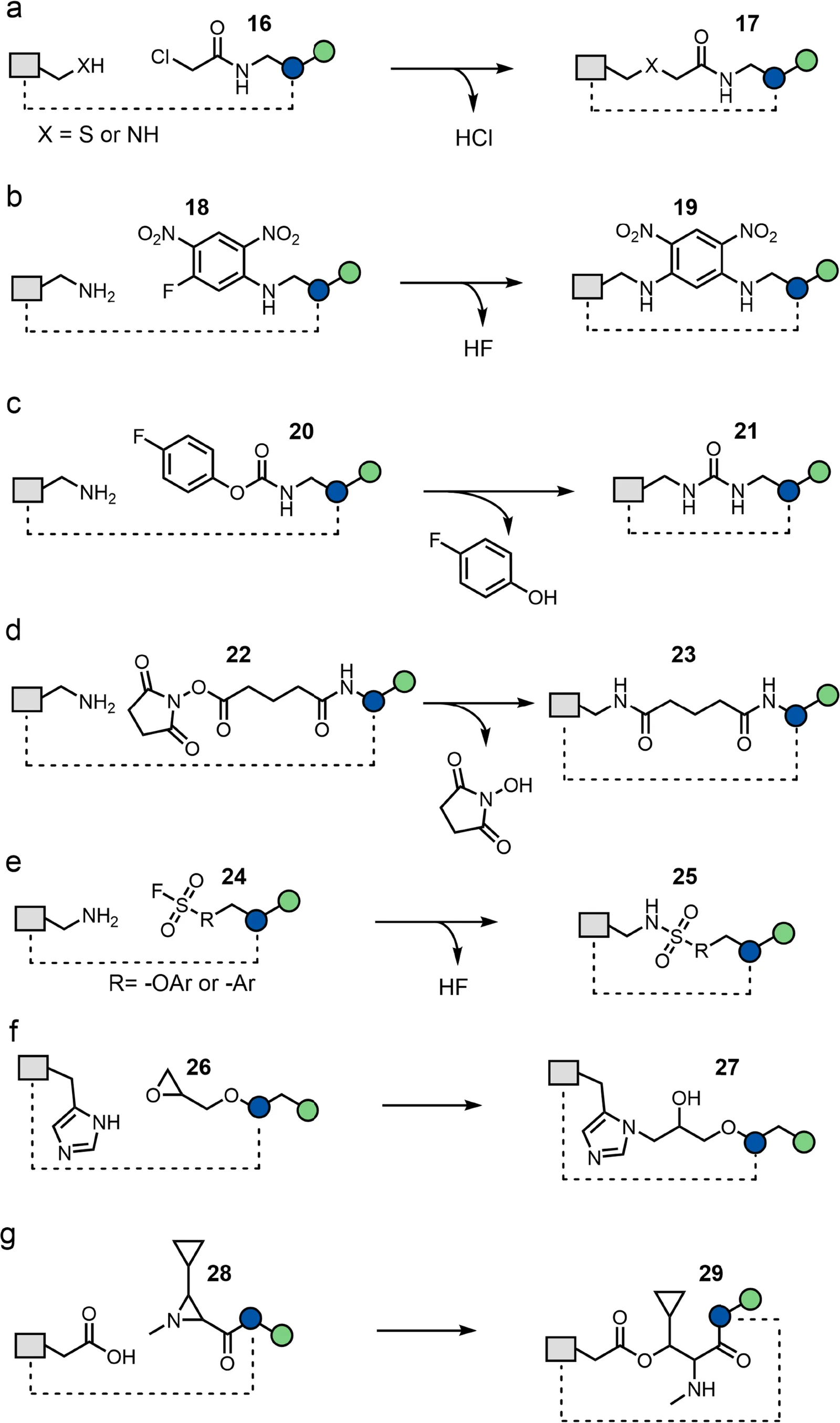
Nucleophilic substitution reactions of (a) an α-chloroacetyl (16), (b) a fluoro-dinitrobenzene (18), (c) a fluoroaryl carbamate (20), (d) an activated NHS ester (22), (e) a sulfonyl fluoride (24), (f) an epoxide (26) and (g) an aziridine (28).

Chloroacetyl groups have also been applied to selectively label a cysteine residue in the globular protein domain KIX from human CBP (CREB binding protein) using the transactivation domain of mixed lineage leukemia as ligand.^34,39^ Molecular dynamics simulations guided the optimization of linker lengths and reactive-group placement, allowing efficient labeling in living cells and the programmed translocation of target proteins from the cytosol to endomembrane compartments. In related work, Wang and colleagues extended the same cysteine-α-chloroacetyl strategy to map the binding interactions between the Src homology 2 (SH2) domain and phosphotyrosine peptides.^56^ Analysis of cross-linked products revealed a reverse binding mode for several phosphopeptides, a conclusion supported by extensive long-timescale simulations.

Beyond alkyl halides, nucleophilic aromatic substitution (SNAr) provides a powerful entry into ligand-directed covalent modification. The electrophile 1-fluoro-2,4-dinitrobenzene (18, FDNB) reacts readily with lysine side chains to afford highly stable arylated products (19, Fig. 5b). FDNB chemistry has been adapted for selective modification of endogenous immunoglobulins under mild conditions, generating homogeneous conjugates suitable for antibody-drug conjugate development.^57^ More recently, Zhang and coworkers applied FDNB-guided lysine substitution to the Csk SH3 domain, achieving >80% conjugation at engineered positions.^43^

Other fluoroaryl electrophiles have similarly been adapted for proximity-directed SNAr reactions. A fluoroaryl-lysine (20) conjugation strategy enabled site-specific labeling of human and mouse antibodies without requiring recombinant engineering (Fig. 5c).^58^ Conjugation yields exceeded 95% across multiple IgG subclasses, supporting efficient delivery of small-molecule fluorophores and other analytical tags. NHS esters (22) provide another classical electrophile for proximity-enhanced nucleophilic substitution. The amine of a lysine side chain can attack an activated carbonyl from an NHS ester to form a stable amide bond (23). Although NHS esters are intrinsically reactive toward primary amines, ligand-directed positioning can confer selective protein modification even in the presence of competing proteins. However, their relatively high intrinsic reactivity can limit selectivity in highly complex biological environments. Kishimoto and colleagues demonstrated that an NHS-bearing affinity peptide could modify native IgG antibodies among purified serum proteins (IgA and human serum albumin), while preserving antigen binding and Fc receptor recognition (Fig. 5d).^59^ This reactivity has since been extended to construct bispecific antibodies and ADCs, underscoring its modularity in protein conjugation workflows.

Sulfur(v) fluoride exchange (SuFEx) chemistry expands the substitution toolbox offering highly stable yet tunable electrophiles.^60,61^ The Rullo lab embedded SuFEx handles (**24**) into bifunctional peptides derived from glycoprotein D of the herpes simplex virus equipped with a tumor antigen-binding molecule to generate covalent antibody-recruiting molecules and to modify glycoproteins on herpes simplex virus (Fig. 5e).^62^ These conjugates enabled targeted recruitment of antiviral antibodies to cancer cells and exhibited irreversible labeling in biochemical assays, providing enhanced immunotherapeutic performance.

Epoxides constitute a mechanistically distinct class of electrophiles whose high ring strain enables nucleophilic opening by histidine, cysteine or lysine. Chen and coworkers applied this reactivity to protein labeling using human carbonic anhydrase II (hCA II) and its well-known ligand benzenesulfonamide.^63^ With only two equivalents of the epoxide probe (26), >90% labeling was achieved, selectively targeting a single histidine residue outside the active site (27, Fig. 5f). The method performed robustly in protein mixtures and cellular extracts, outperforming several alternative electrophiles such as diazoketones and benzyl chlorides. Hamachi and coworkers later employed epoxide chemistry in a one-pot sequential labeling and hydrazone/oxime exchange workflow, enabling selective modification of two histidine residues by reopening of the active site of hCA II to restore enzyme activity.^64^ Takaoka and colleagues further extended epoxide-embedded ligands to living cells, showing that only probes oriented toward the FKBP12 binding pocket yielded efficient covalent labeling.^65^ The labeling was assessed by gel/blot readouts without residue-level identification.

A clinically relevant example is zoldonrasib, an orally bioavailable covalent inhibitor of oncogenic KRAS G12D that features a strained aziridine (28) capable of undergoing nucleophilic ring opening by the carboxylate side chain of an aspartic acid (29, Fig. 5g).^66^ Rather than relying on the ligand alone to achieve selectivity, zoldonrasib first engages intracellular cyclophilin A, forming a ternary complex with KRAS G12D that positions the otherwise low-reactivity aziridine for covalent capture of Asp12. This proximity-driven mechanism enables selective covalent engagement of KRAS G12D and is currently evaluated in clinical trials for KRAS G12D-mutant cancers, including non-small cell lung cancer and pancreatic ductal adenocarcinoma.

The electrophiles discussed in this section differ considerably in their intrinsic reactivity. Chloroacetamide-type electrophiles are well established in activity-based protein profiling as broadly reactive, relatively non-selective probes that, at moderate concentrations, react readily with accessible cysteines in the absence of a targeting ligand.^67^ The epoxide probe 26, by contrast, is stable toward proteins in solution and requires precise positioning for conjugation.^63^ Beyond conventional protein labeling, several of the electrophiles introduced above have also been employed for an additional purpose: ligand-directed probes bearing acrylamide, chloroacetamide, or epoxide groups have been used to covalently attach reversible photoswitches onto native, endogenous ion channels, without the need for genetic engineering.^68^

#### 2.1.4 Electrophilic aromatic substitutions

Electrophilic aromatic substitution offers a distinctive route to ligand-directed covalent labeling by installing an electrophilic aromatic moiety within the ligand that can undergo selective coupling with nucleophilic residues. A first demonstration of this concept was reported by the Xia group, who developed a deactivated aryldiazonium functionality incorporated into a synthetic peptide ligand for selective labeling of soluble SH3 proteins (Fig. 6).^69^ The ligand carried a phenylalanine analog bearing an aryldiazonium group (30), which reacted selectively with tyrosine residues (31) while remaining inert to non-proximal residues. This selectivity arose from careful modulation of the electronic properties of the aryl ring, ensuring that the diazonium group remained sufficiently reactive only when properly oriented through ligand binding. Given the prevalence of tyrosine residues in SH3 binding pockets, this approach presents a promising strategy for developing covalent affinity probes for SH3 domains.

**Fig. 6:**
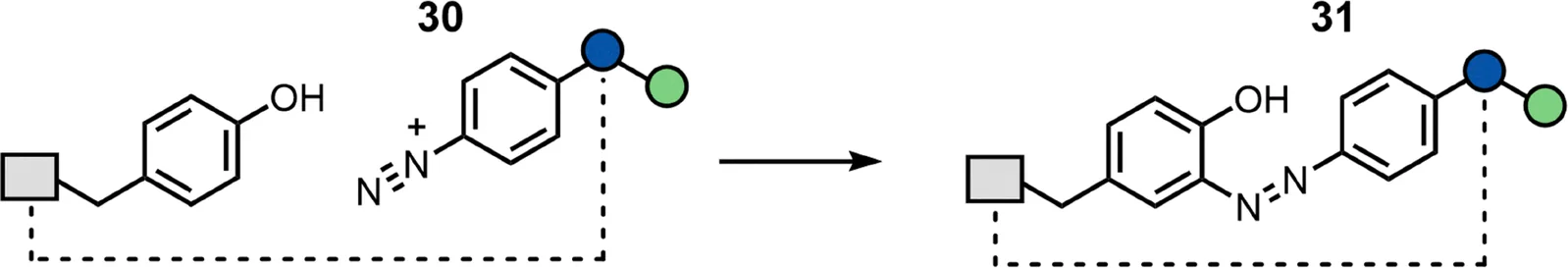
Electrophilic aromatic substitution with an aryl diazonium (30).

These studies were conducted on isolated, soluble proteins, leaving open the question of whether such affinity-mediated azo coupling could operate effectively in more complex biological environments, including membrane proteins in living cells. This question was addressed by the Checco group, who advanced this methodology by demonstrating that aryl diazonium-bearing peptide analogs can covalently label G protein-coupled receptors on the surfaces of living cells.^70^ Using fluorescein- or biotin-tagged analogues of neurotensin and exendin-4, they achieved selective covalent labeling of NTSR1 and GLP1R without genetic modification of the receptors, enabling both live-cell imaging and proteomic enrichment. Labeling was strictly sequence-dependent and abolished by competition with the native ligand, confirming that covalent modification occurs only within the receptor binding pocket. Although azo coupling typically targets tyrosine, and, to a lesser extent, histidine residues, the specific amino acid(s) modified in these receptors were not identified and remain to be mapped.

#### 2.1.5 Condensation reactions

Condensation-based strategies exploit reversible imine formation between aldehyde-bearing ligands and nucleophilic residues to achieve proximity-dependent capture under mild conditions. An early example was reported by Siegel and coworkers, who designed gluten-peptide analogues containing an α-amido aldehyde (32) oriented toward Lys-β71 in the HLA-DQ2 binding groove (Fig. 7a).^71^ Upon ligand binding, the aldehyde and lysine were brought into close proximity, enabling reversible imine formation (33), most likely involving the ε-amine of Lys-β71, although the adduct could not be directly observed. Functionally, this transient covalent capture stabilized the peptide-MHC complex, increasing affinity at neutral pH and extending the dissociation half-life more than eightfold relative to the unmodified peptide.

**Fig. 7:**
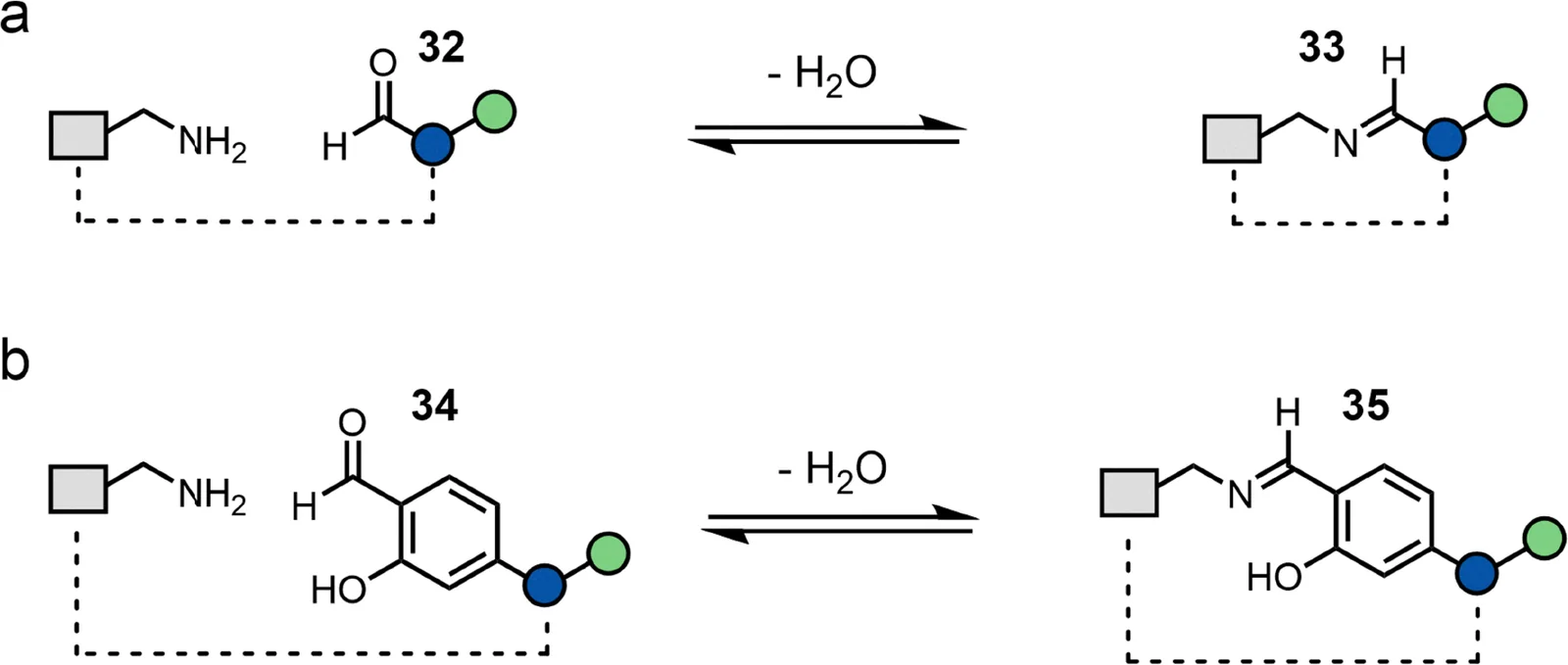
Condensation reactions with (a) an α-amido aldehyde (32) or (b) a 2-hydroxybenzaldehyde (34).

Dal Corso and colleagues later explored benzaldehyde-mediated lysine capture across proteins with diverse lysine content, including targeting human serum albumin, CA II and human interleukin-2.^72^ Their ligands incorporated a 2-hydroxybenzaldehyde unit (34), a fluorescein tag and a 12-bp locked-nucleic-acid element. The *ortho*-hydroxybenzaldehyde facilitated reversible imine formation with lysine side chains (35), stabilized by intramolecular hydrogen bonding (Fig. 7b). Although individual lysine residues were not mapped, hydroxylamine quenching and structural modeling strongly supported reversible lysine imine formation as the key interaction. A more detailed analysis of benzaldehyde-based lysine modification, including design features of salicylaldehydes for covalent drug targeting, is provided in a comprehensive review by the Dal Corso group.^73^

#### 2.1.6 Ligand oxidation

Ligand oxidation-mediated ligation represents an early example of a permanent ligation strategy followed by subsequent cleavage of the ligand. This approach was reported by Hamachi and coworkers, who designed polysaccharide-based fluorescent biosensors targeting the lectin concanavalin A (Con A).^74^ In their system, an α-d-mannoside ligand positioned a saccharide-bearing fluorophore (36) within the Con A binding pocket. Oxidation of the saccharide moiety with periodate generated free aldehyde groups on the polysaccharide backbone (38, Fig. 8). This oxidation renders the polysaccharide susceptible to degradation under physiological conditions, which may be relevant for drug-delivery applications, and enables subsequent chemical derivatization. The newly formed aldehydes were then subjected to reductive amination with a hydrazine-functionalized fluorophore (39) to yield a covalent Con A adduct (40). Proteolytic digestion followed by MALDI-TOF analysis showed that modification occurred selectively at tyrosine residues within the binding pocket. Importantly, this modular strategy allows tuning of carbohydrate specificity by exchanging saccharide ligands and pairing them with their corresponding lectins, and while not entirely removing the ligand, it inactivates its binding capabilities.

**Fig. 8:**
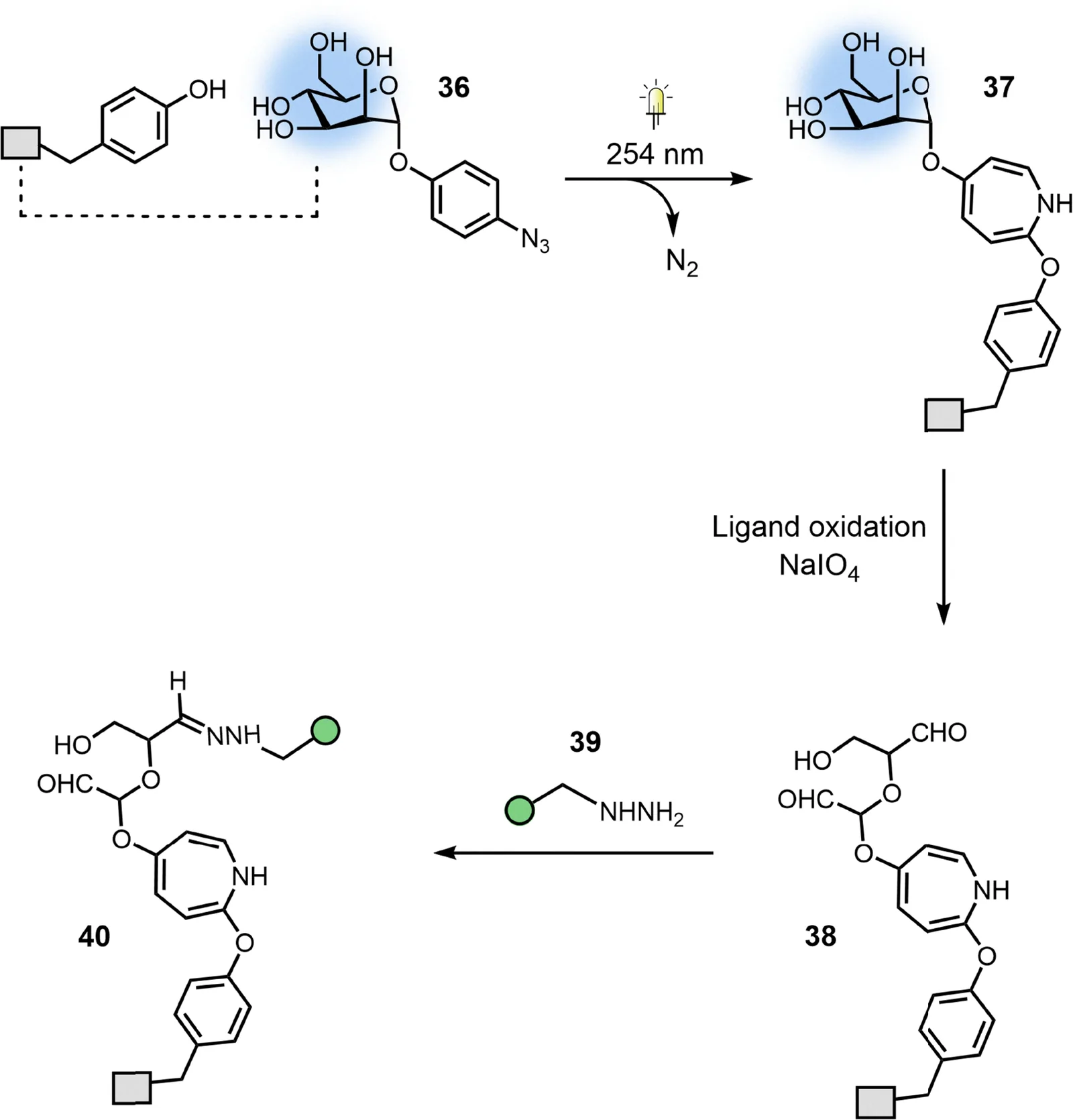
Ligand oxidation reaction with a saccharide-based ligand (36).

### 2.2 Ligation and subsequent cleavage

For many labeling strategies it is not desired to retain a ligand attached to the protein of interest. This motivated the design of ligation reactions which are followed by selective cleavage thereby allowing the installation of covalent modifications while removing the ligand in a subsequent step. These approaches proceed through an affinity-guided reaction that anchors a reactive handle at a specific residue, after which controlled cleavage releases the ligand or unmasks a functional group for downstream conjugation. This two-step logic offers temporal control, minimizes steric perturbation from the initial recognition element, and supports traceless or semi-traceless protein modification.

#### 2.2.1 Disulfide cleavage

Disulfide linkages offer a built-in cleavage motif whose stability and reactivity depend strongly on the redox environment. Hamachi and co-workers applied this concept in an early post-photoaffinity labeling workflow using the lectin Con A (Fig. 9a).^75^ A saccharide-based ligand containing a diazirine and a cleavable disulfide (41) was first photolinked to Con A (42). Reduction with dithiothreitol (DTT) then cleaved the disulfide, releasing the ligand (43) and generating a free thiol (44, SH-Con A). This newly exposed cysteine was subsequently functionalized with an iodoacetylated dansyl fluorophore (45, IAEDANS-Con A), producing a fluorescent lectin (46) whose emission intensity decreased upon addition of free methyl-α-mannoside, enabling direct monitoring of ligand binding. The same group later expanded this chemistry in a broader post-photoaffinity-labeling modification (PPALM) platform, which systematically investigated amino-acid specificity, labeling sites, and saccharide-dependent fluorescence responses across a diverse set of lectins.^76^ Full analysis revealed that the modification occurred with a tyrosine residue.

**Fig. 9:**
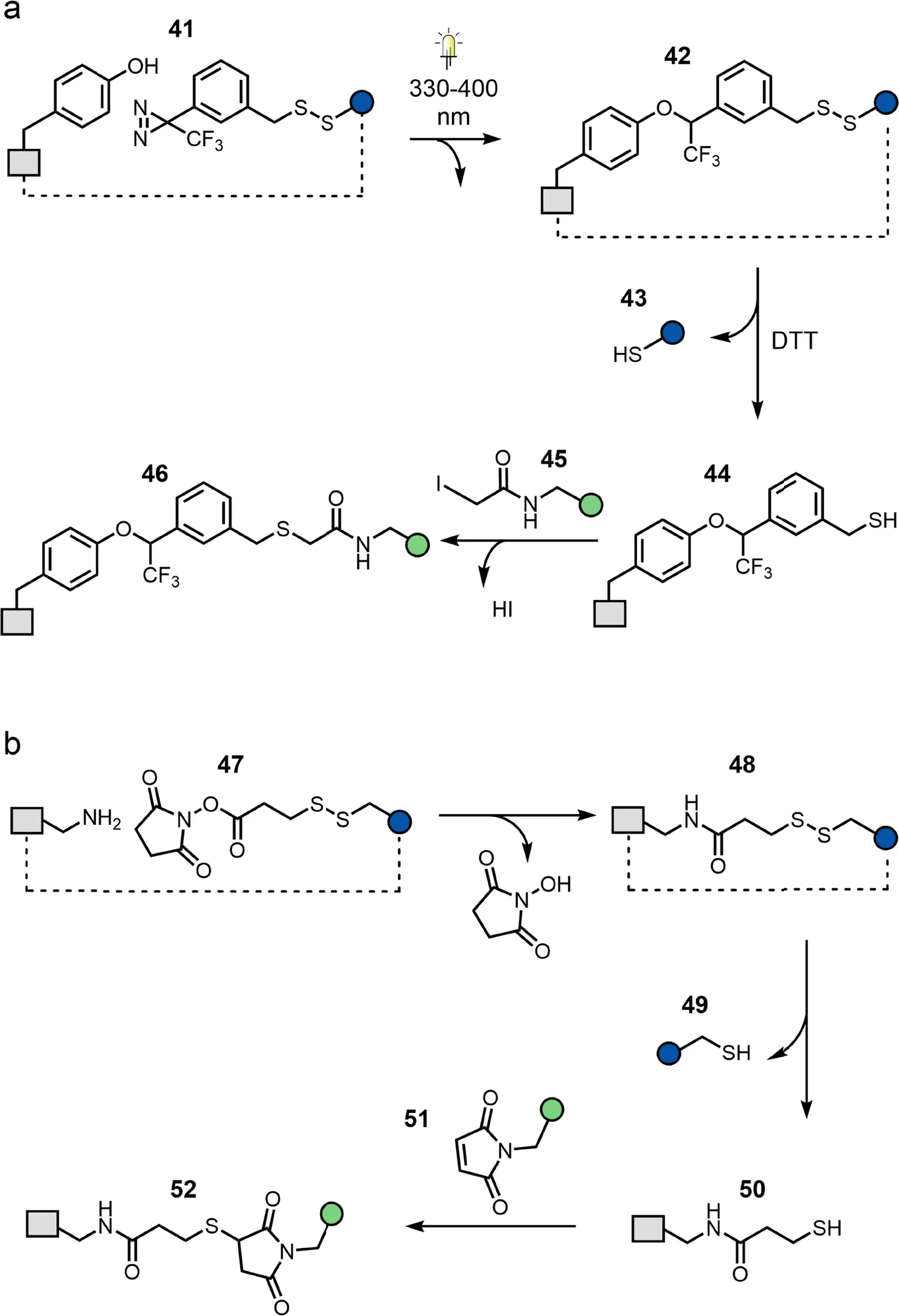
Disulfide cleavage reactions with (a) a diazirine (41) which is photolinked to the protein (42), or (b) an NHS ester (47).

More recently, Yamada and colleagues applied a cleavable disulfide linker to the design of regiodivergent antibody-drug conjugates from native antibodies (Fig. 9b).^77^ Their method relies on an Fc-binding affinity peptide that delivers an NHS ester (47) to a specific lysine residue (48). Subsequent reduction cleaves the disulfide that connects the peptide (49) to the installed electrophile, generating a free thiol on the antibody (50). This thiol then reacts with maleimide-functionalized moieties (51) to assemble the final conjugate (52). Although the approach affords high site selectivity and improved anti-tumor activity relative to native trastuzumab, the multistep ligand-delivery and release sequence highlights the practical challenges of achieving clean traceless modification.

#### 2.2.2 Photocleavage

Amide bond formation is a robust modification strategy owing to the high stability of the resulting linkage, yet appropriately designed systems permit selective cleavage or exchange under defined conditions. The Bode lab exploited this concept using a high-affinity Fc-binding peptide to position an NHS ester adjacent to Lys246/248 of native IgG antibodies, producing site-specific lysine acylation without requiring antibody engineering (Fig. 10).^78^ The peptide incorporated a photocaged hydroxylamine (53), enabling a two-step sequence: proximity-guided NHS ester reaction installs the hydroxylamine on the Fc-proximal lysine (54), and subsequent UV irradiation removes the protecting group to liberate a free hydroxylamine on the antibody surface. This reactive handle then undergoes rapid potassium acyltrifluoroborate ligation with quinolinium acyltrifluoroborates (55) at physiological pH, yielding a stable amide bond (57) while releasing the affinity peptide (56). Mass spectrometry and MS/MS mapping confirmed exclusive modification at Lys246/Lys248, demonstrating that orientation within the Fc pocket dictates selectivity in this amidation-cleavage sequence.

**Fig. 10:**
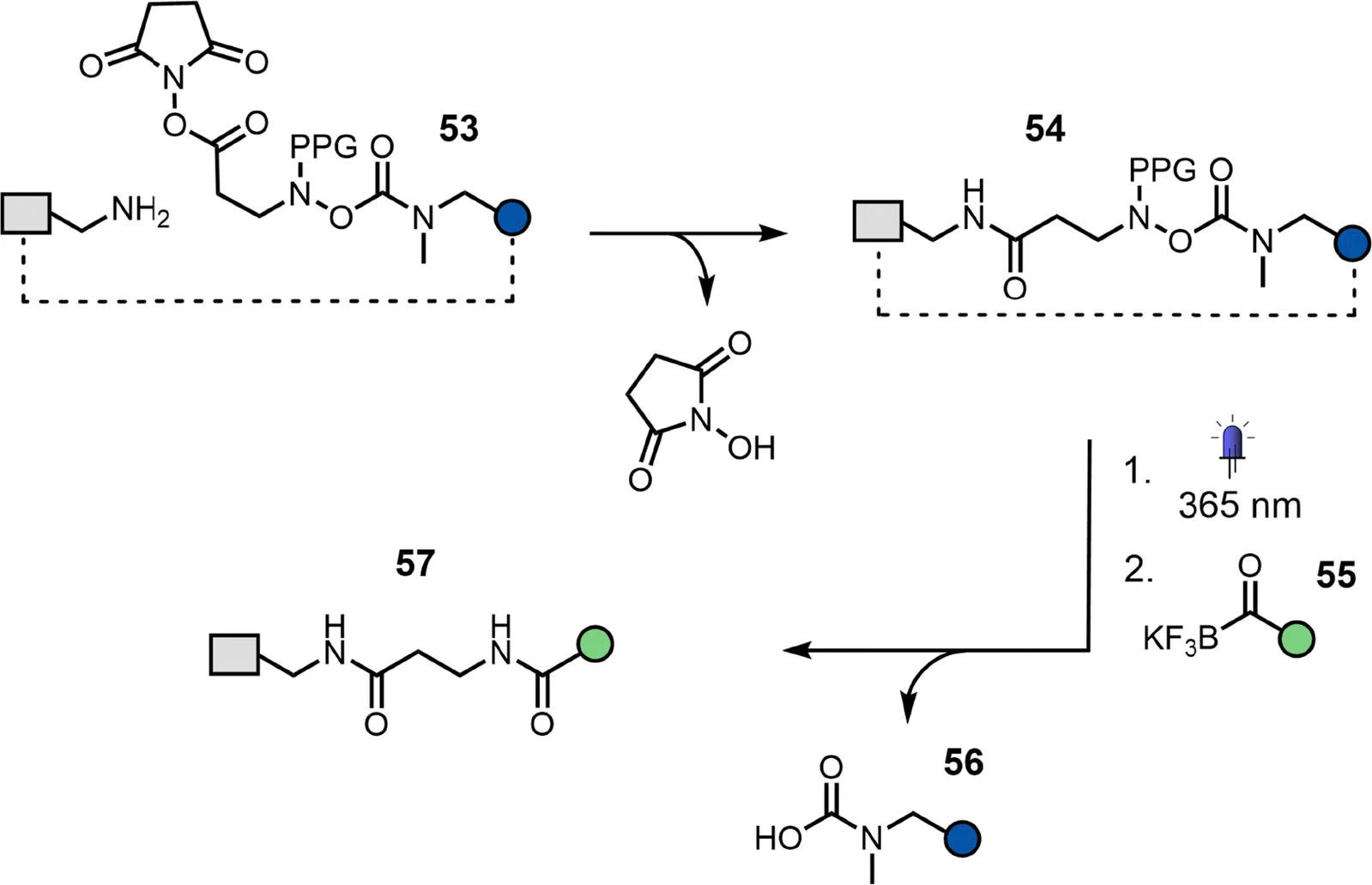
Photocleavage reaction with a photocaged hydroxylamine (53) followed by potassium acyltrifluoroborate ligation (57).

## 3. Transfer reactions

Transfer reactions involve proximity-induced reactions in which a ligand-tethered functional moiety is transferred to a protein resulting in the release of the ligand. In contrast to the ligation reactions, the ligand can dissociate from the protein after the modification event, allowing for traceless, site-specific modifications. Transfer reactions can be divided into three categories based on their mode of transfer (Fig. 11) involving direct transfer, copper-assisted diazo transfer, and photo-induced release and transfer.

**Fig. 11:**
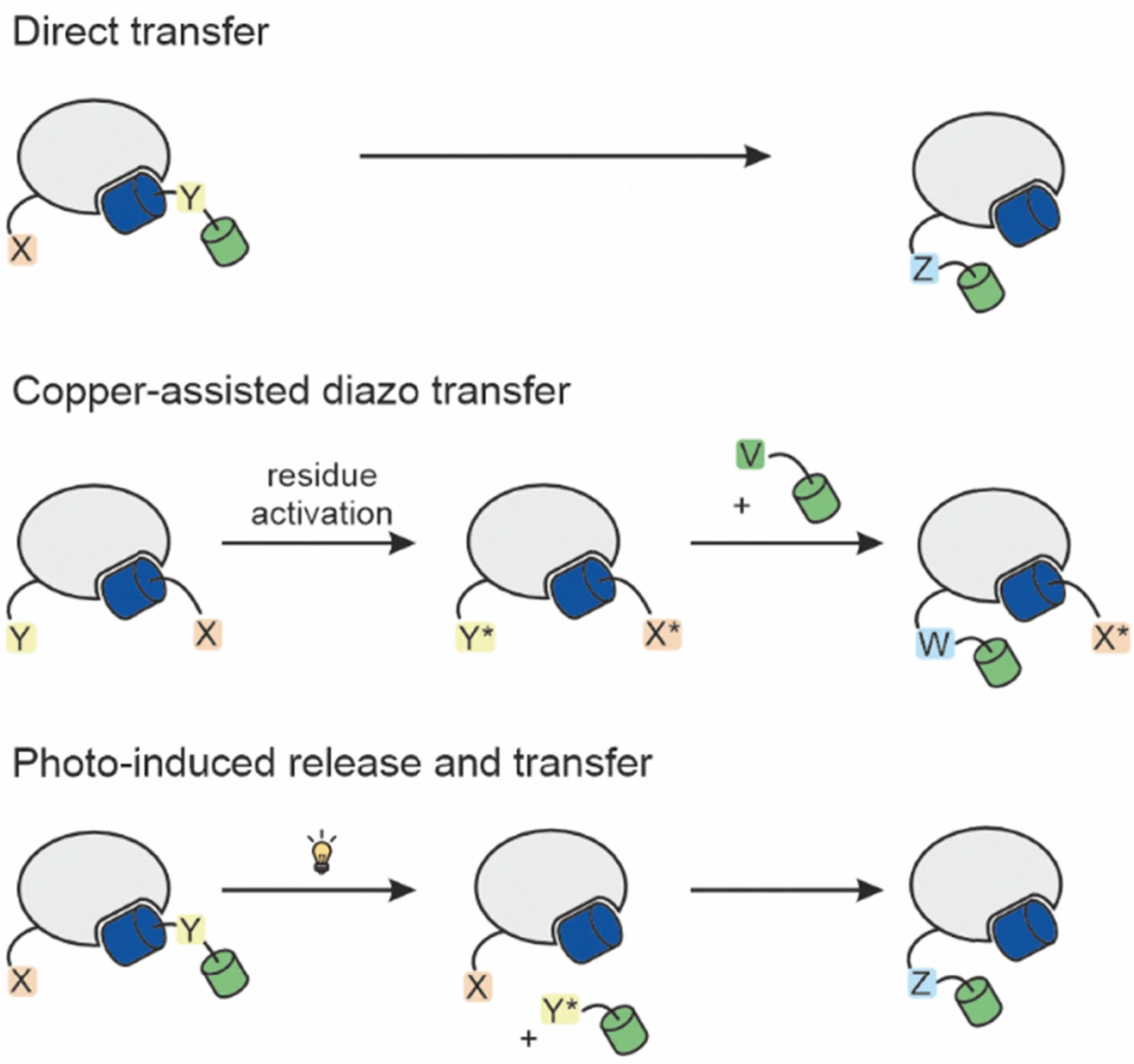
Overview of different transfer strategies involving direct transfer (top), copper-assisted diazo transfer (middle) and photo-induced release and transfer (bottom).

### 3.1 Direct transfer

Direct transfer reactions refer to the traceless proximity-induced transfer of a functional moiety involving a concomitant ligand release. The protein ligand is typically linked to the functional moiety *via* an electrophilic group which reacts with nucleophilic residues upon binding to the target protein. Given the diversity of electrophilic reactivities used for direct transfer reactions, these were categorized as follows: (i) alkyl and alkynyl transfer, (ii) oxycarbonyl transfer, (iii) acyl transfer, (iv) sulfonyl transfer, (v) nucleophilic aromatic substitution and (vi) conjugate addition.

#### 3.1.1 Alkyl and alkynyl transfer

Protein modifications which involve an alkyl or alkynyl transfer typically occur *via* a nucleophilic substitution reaction or an initial Michael addition. These ligand-directed transfer reactions employ a diverse set of leaving groups and were pioneered by the Hamachi group.^2,11^ One of the first electrophiles investigated were tosylates/phenylsulfonates esters, the so-called ligand-directed tosyl chemistry (LDT, Fig. 12a).^79–85^ Through a nucleophilic substitution reaction the electrophilic tosylate (58) transfers a methylene group on the protein together with the functional moiety (60). Subsequently, the ligand is released, carrying the tosylate ion leaving group (59). In the initial experiments using this chemistry, a fluorophore, biotin tag or ^19^F-NMR probe were successfully transferred onto a carbonic anhydrase (CA) using its ligand benzenesulfonamide.^79^ It was observed that only one histidine was modified and that protein function was not lost after the modification.^79^ Notably, labeling was also achieved in living mice, demonstrating the selectivity and broad applicability of this chemistry.

**Fig. 12:**
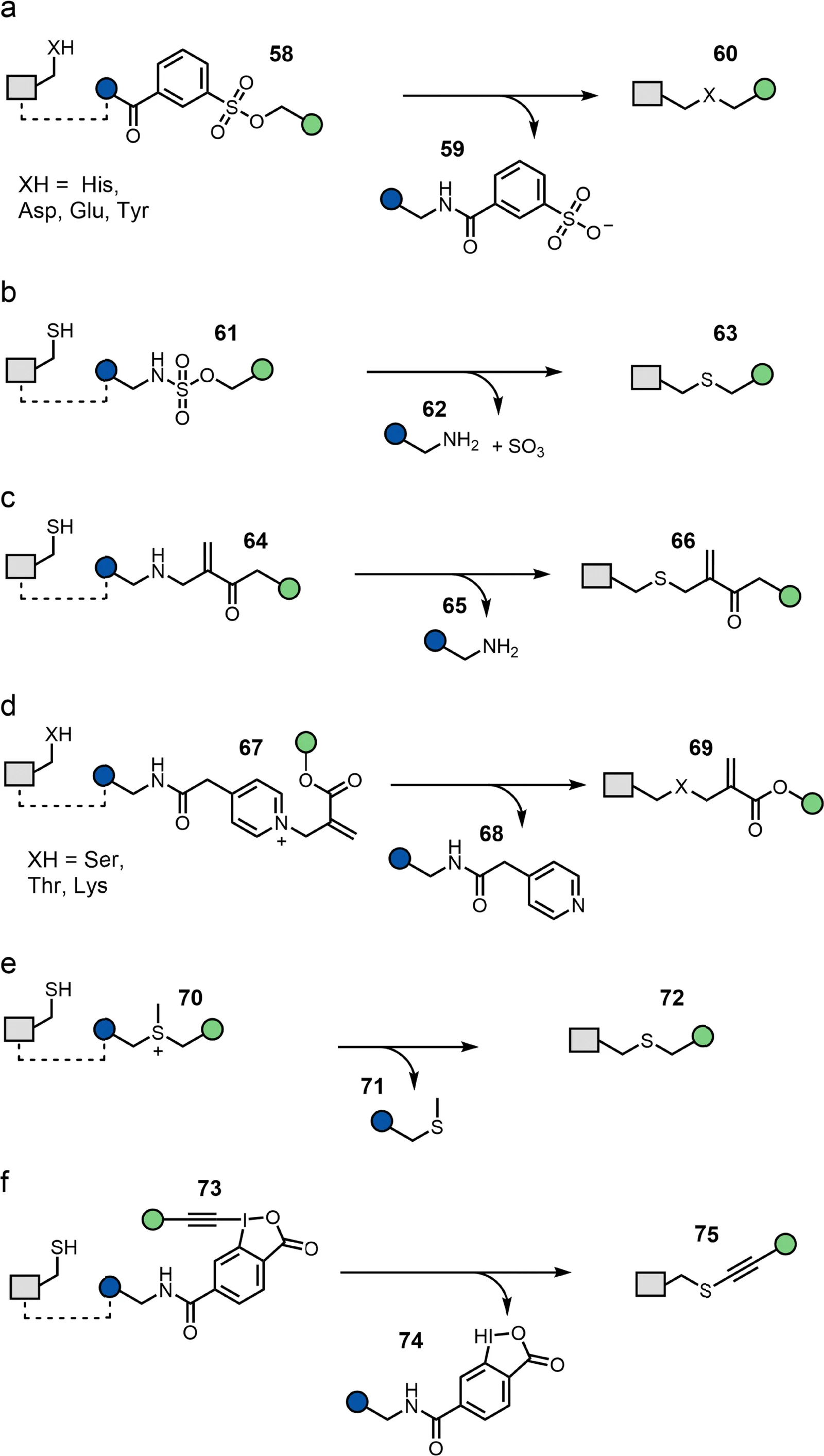
Nucleophilic substitution reactions or Michael additions resulting in alkyl(ynyl) transfer with (a) an aryl tosylate (58), (b) a sulfate acetamide (61), (c) a methacrylamide (64), (d) an α,β-unsaturated carbonyl (67), (e) a sulfonium (70), (f) an alkynylation with hypervalent iodine electrophiles (73).

LDT chemistry has also been exploited to transfer an organic dye to CA II and to a Src homology 2 domain. The ligand was designed to include a fluorescence quencher thereby enabling a ‘turn-on’ fluorescent biosensor for a specific protein.^80^ Similarly to the previous example, only histidine residues were modified. The labeling occurred on the purified target protein as well as in bacterial cell lysate.^80^ Similarly, phosphopeptide-dependent fluorescent labeling with a diterpene fusicocin A ligand containing a tosylate ester group was achieved by the Hamachi group.^86^ Other examples utilizing LDT chemistry have shown modifications of tyrosine, glutamic acid or aspartic acid residues in addition to histidine both *ex cellulo* as well as in live cells.^81–83,87,88^ It was observed that the length and rigidity of the spacer between the ligand and the tosylate plays an important role in the yield and the observed modification site.^81,82^ Notably, labeling with the electrophilic tosylate is slow, as it requires hours to days for completion, representing a limitation in LDT chemistry. More recently Reddi and colleagues utilized a sulfamate acetamide-containing inhibitor (ibrutinib) of BTK (61) to transfer a methyl or propargyl group achieving the specific modification of a cysteine residue of BTK (63) while preserving its enzymatic activity (Fig. 12b).^89^ Using this chemistry, a fluorescent turn-on probe was developed using coumarin as fluorescent moiety showing eventually in cellulo activity.^89^

Using methacrylamide ibrutinib analogues (64), a cysteine in BTK was selectively labeled (Fig. 12c) resulting in the installation of a methacrylamide (66).^90^ An alkyne and fluorophore tag were successfully transferred to the target protein, and protein activity in cells was shown to be preserved after modification.^90^ Alternatively, Kurbanov and coworkers utilized a pyridinium substituted α,β-unsaturated carbonyl moiety (67) to achieve multisite labeling on CA *via* its ligand benzenesulfonamide.^91^ In this case, the pyridinium moiety acts as leaving group (68) facilitating the transfer of the α,β-unsaturated carbonyl group (69, Fig. 12d) to histidine, serine, lysine and threonine. Notably, it was shown that labeling could occur with membrane proteins on live cells.^91^

While above described alkyl transfer reactions employed small molecular ligands, Wang and coworkers have exploited a sulfonium-modified peptide (70) as ligand achieving cysteine-selective modification of a PDZ domain (72, Fig. 12e).^92^ Highest modification efficiencies were observed for benzyl and adamantane substituted sulfonium moieties.^92^ In addition to the above described ligation reaction (Fig. 4b), Waser and coworkers also describe an EBX-based transfer reaction for protein labeling.^41^ Using peptide-tethered silyl-EBX reagents (73), they achieved ligand-directed, traceless cysteine alkynylation (75) of the KEAP1-KELCH domain, installing an alkyne handle at a specific cysteine residue with concomitant release of the peptide ligand (74, Fig. 12f). The resulting alkynylated cysteine could be directly functionalized *via* SPAAC, and labeling remained efficient in protein mixtures and cell lysate.

#### 3.1.2 Oxycarbonyl transfer

Ligand-directed chemistry transferring an oxycarbonyl group to a nucleophilic residue has been extensively investigated. One of the reactions often utilized is the so-called ligand-directed acyl imidazole chemistry designed in the Hamachi group.^93,94^ This process entails a nucleophilic acyl substitution reaction of an alkyloxyacyl imidazole (76), resulting in the transfer of an oxycarbonyl group onto the protein (78) and the subsequent release of the acyl imidazole (77) attached to the ligand (Fig. 13a).^93^ The Hamachi group has demonstrated the use of this chemistry for studying the biology of membrane-bound receptors through live cell labeling.^93,95–100^ As a proof of concept, acyl imidazole chemistry was demonstrated by selectively labeling a lysine residue in proximity to the binding pocket of dihydrofolate reductase (DHFR) by using methotrexate as the affinity ligand.^93^ The same labeling strategy was applied to membrane bound folate reductase (FR) in live cells, where it exhibited a 12-fold increase in efficiency compared to labeling using LDT chemistry (Fig. 12a).^93^ This methodology was applied to various membrane proteins,^98,99^ and has also been used to design semisynthetic fluorescent biosensors for GABA_A_ receptors on live cells.^97^ Notably, labeling in live neuron cells with a fluorophore or biotin of AMPA receptors was achieved.^96^ In addition to lysine, hydroxyl-containing residues such as serine and tyrosine may in principle also be targeted by acyl imidazole chemistry.^94^ In more recent work from the Hamachi group, labeling of endogenous neurotransmitter receptors was successfully explored in living mouse brains.^95^ Furthermore, acyl imidazole chemistry was applied in the design of affinity probes for labeling of matrix metalloproteases.^101^

**Fig. 13:**
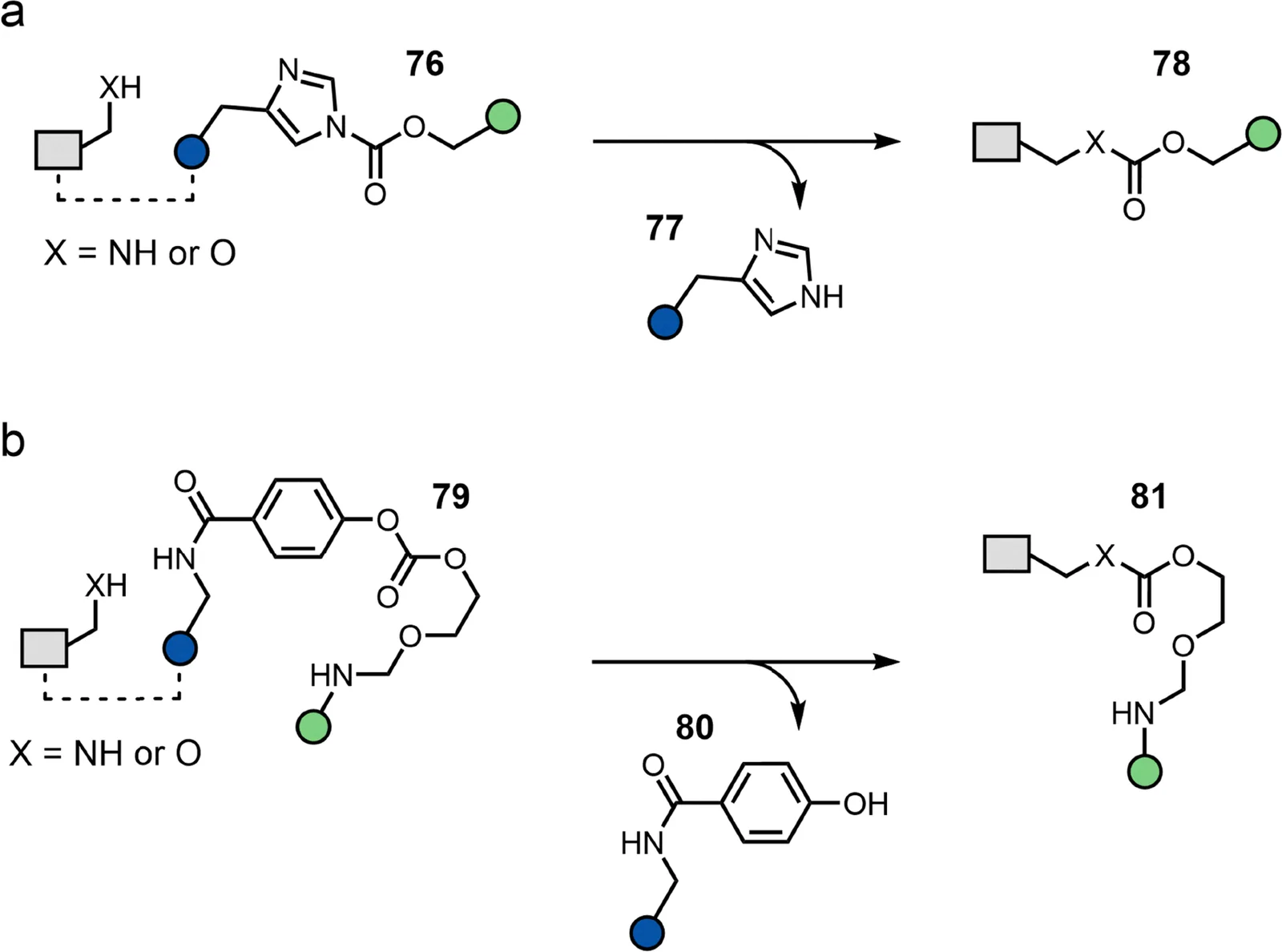
Nucleophilic acyl substitution reactions resulting in oxycarbonyl transfer *via* an (a) acyl imidazole (76) or (b) a carboxyphenyl (79).

Acyl imidazole chemistry has also been combined with click chemistry to attach a functional moiety onto an AMPA receptor in neuronal culture conditions, utilizing the AMPA-selective ligand PFQX making it a bioorthogonal two-step labeling strategy.^100^ This did not affect the viability of the neurons, in contrast to earlier methods used to label AMPA receptors. The Sames lab utilized the acyl imidazole chemistry for AMPA receptor modification with the same PFQX ligand for the successful chemical targeting of voltage sensitive dyes in specific neuronal cell types in brain tissue.^102^ Furthermore, acyl imidazole reactivity has been employed for labeling of metal-binding proteins,^103^ where an azide handle was conjugated to a lysine residue allowing further functionalization using click chemistry. A recent example describes the generation of covalent immune recruiters for immune recognition.^104^ Here, the reactive acyl imidazole is placed in between an antibody recognition element and a target binder, which were desthiobiotin and glutamate urea ligands. Upon binding to an anti-DNP IgG, a single modified lysine was identified. Labeling occurred successfully in human serum, showcasing a new design for future developments of antibody engagers or antibody recruiting molecules.^104^

Oxycarbonyl transfer was also achieved by using a ligand with a carboxyphenyl group (79) (Fig. 13b).^105^ Here, an Fc domain-binding peptide was used as ligand to bind an antibody (Trastuzumab) facilitating fluorophore labeling.^105^ This strategy was extended to target two lysine residues in proximity of the binding site of the peptide used to bind to Trastuzumab (81).^106^ Various conjugates were designed with the most effective candidate being able to transfer radionuclide chelators, fluorescent dyes, click-chemistry reagents, pre-targeted imaging reagents and cytotoxic small molecules to the target antibody in high yields.^106^

#### 3.1.3 Acyl transfer

As an alternative strategy, the ligand-directed transfer of acyl moieties has been applied for site-specific protein modifications (Fig. 14). In a target elucidation study, a phenyl ester group (82a) was employed to transfer an acyl group onto actin (84), with a phenolate acting as leaving group (83, Fig. 14a).^107^ This resulted in the modification of a specific lysine residue and the transfer of an immunoaffinity fluorescent tag. In this context, it was shown that ligand-directed labeling was more specific and efficient than non-proximity-guided protein labeling.^107^ Similar chemistry was applied to site-selectively label the Fc domain of an immunoglobulin.^108^ A peptide containing a thioether was used as ligand and MS analyses revealed specific modification of a lysine residue near the peptide binding site. The transfer of an azide, alkyne, fluorophores, and biotin was demonstrated *via* the acyl substitution reaction. This included the construction of a bispecific antibody complex with potential for antibody-based immunotherapies.^108^

**Fig. 14:**
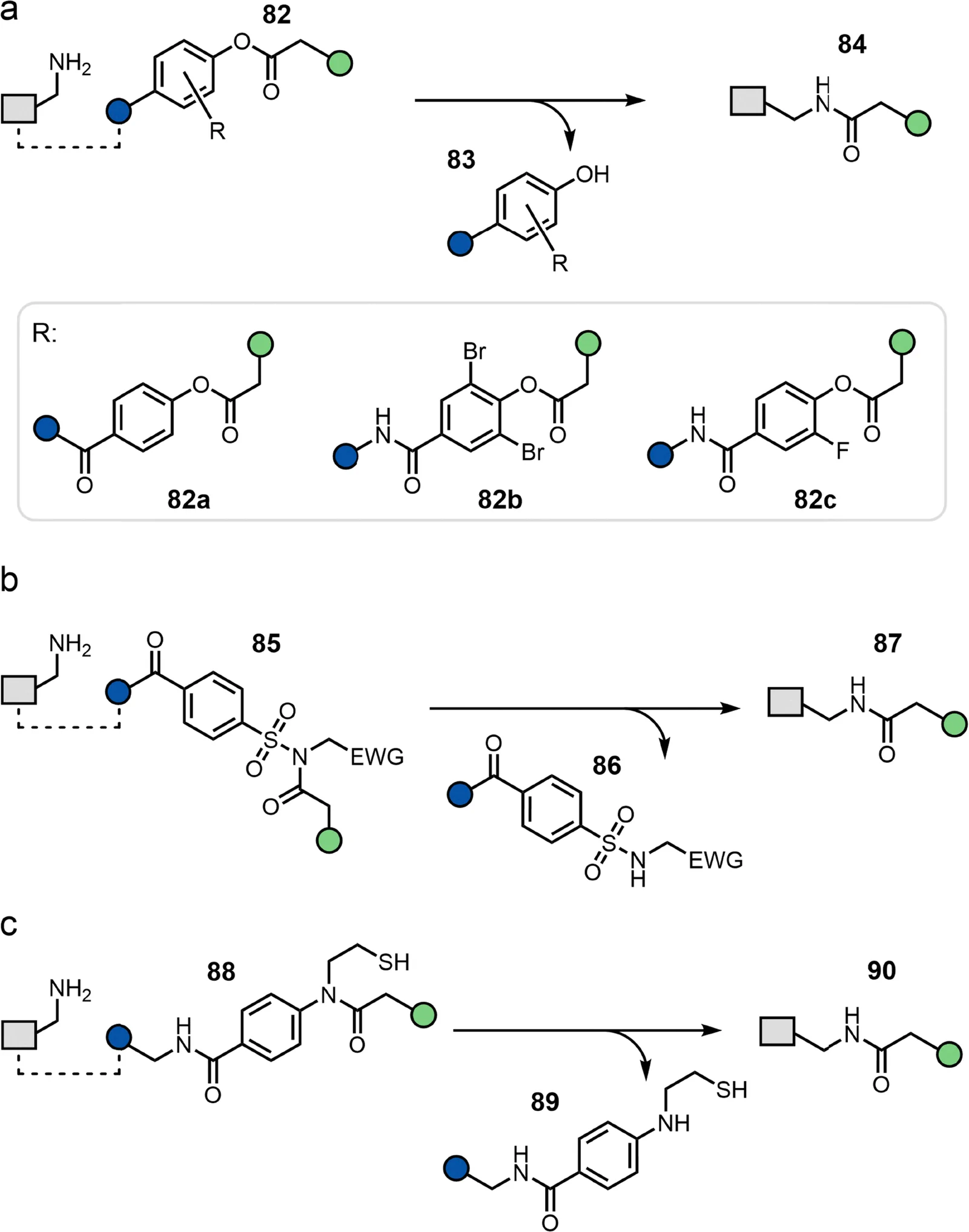
Nucleophilic acyl substitution reactions resulting in acyl transfer *via* (a) a phenyl ester group (82a–c), (b) an *N*-acyl-*N*-alkyl sulfonamide (85) or (c) *N*-sulfanylethylanilide (88). EWG = NO_2_ or CN.

The Hamachi group demonstrated the use of derivatives of phenyl esters for the acylation of proteins.^109^ After optimization of the reactivity and stability, dibromophenyl benzoate (82b) was identified as a suitable reactive group. Here, an *ortho*-dibromophenyl ester is used for the nucleophilic acyl substitution with dibromophenolate acting as leaving group (Fig. 14a). This chemistry was applied for the labeling of CA and DHFR, both in biochemical assays as well as in live cells, utilizing their well-known ligands benzenesulfonamide and trimethoprim respectively. The modification site was identified to be predominantly a lysine near the binding site of the ligand, but also modification of a histidine was observed. Various fluorophores were successfully appended to the protein, highlighting the use for imaging of proteins in live cells.^109^ In more recent work, a fluoro-substituted phenyl ester (82c) was utilized for the labeling of an adenosine A_1_AR receptor in live cells (Fig. 14a).^110^ After modification of a single lysine, fluoro-substituted phenol attached to the ligand was released.^110^

In the pursuit of rapid labeling techniques in live cells, the Hamachi group explored novel ligand-directed chemistries. They selected *N*-acyl-*N*-alkyl sulfonamide (NASA) derivatives (85) as promising electrophilic reactive groups in acyl transfer reactions (87, Fig. 14b).^11,111^ Notably, the labeling rate using NASA derivatives exceeded that of labeling with tosylate, acyl imidazole and dibromobenzoate chemistries. Various electron-withdrawing groups were tested at the *N*-alkyl position of NASA, given the dependence of the *N*-acyl group's electrophilicity on the *N*-alkyl group. The labeling reaction was tested in target proteins FKBP12 with its SLF ligand and in *Escherichia coli* DHFR with trimethoprim, revealing the cyano group as the optimal *N*-alkyl substituent. In both proteins, a single modified lysine was identified near to the binding site, indicating a preference for lysine residues. Kinetic analyses underscored the significance of the ligand binding affinity for labeling kinetics, highlighting that NASA chemistry requires a ligand with sub-micromolar affinity for efficient and rapid labeling.^111^ It was also found that the NASA probe had higher labeling yields compared to the corresponding acyl imidazole derivative showing specificity towards lysine. More recently, Io and Wang have utilized NASA chemistry to develop a two-step ligand-directed functionalization strategy.^112^ First, protein labeling occurs *via* ligand-directed NASA chemistry installing a clickable tag on the protein which can then be further functionalized. Interestingly, their findings revealed three distinct lysine modification sites. In another attempt, Denda and colleagues utilized the electrophilic *N*-sulfanylethylanilide (88) for an N–S acyl transfer acylation (90) on CA and cyclooxygenase 1 (Fig. 14c).^113^ The anilide is activated only upon conversion into the corresponding thioester which is triggered by the presence of the acidic and basic residues of the protein. Selective labeling in a protein mixture was achieved.^113^

#### 3.1.4 Sulfonyl transfer

In addition to the already described ligand-directed modification reactions, the Hamachi group pursued approaches aimed at enhancing efficiency and selectivity. Sulfonylation of CA was achieved using an *N*-sulfonyl pyridone moiety (91) conjugated to the ligand benzenesulfonamide (Fig. 15).^114^ This modification resulted in the labeling of lysine and tyrosine residues (93) in close proximity to the binding pocket. Remarkably, a labeling yield of 100% was attained after 12 h, demonstrating increased reaction rates compared to the previously developed LDT and acyl imidazole chemistries.^114^

**Fig. 15:**
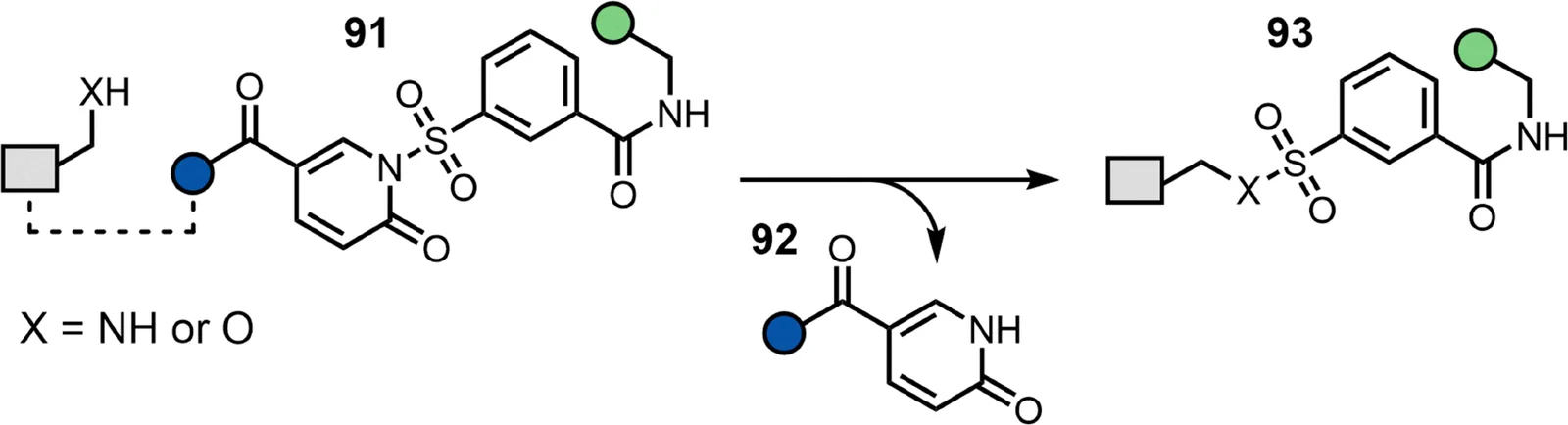
Sulfonylation *via* an *N*-sulfonyl pyridone (91).

#### 3.1.5 Nucleophilic aromatic substitutions

A nucleophilic aromatic substitution with a modified nitrobenzoxadiazole (94) was employed to modify a lysine in avidin, both in buffer and cell lysate environments (Fig. 16a).^115^ This strategy aimed to develop a specific “turn-on” fluorescent probe using a hydroxy-modified nitrobenzoxadiazole derivative (94) as non-fluorescent precursor. Upon reaction with the primary amine of the lysine side chain, amino-nitrobenzoxadiazole (96) is formed exhibiting increased fluorescence. In an alternative approach, Otsuki and coworkers utilized 5-sulfonyl tetrazole conjugated to ligand cyclosporine A (97) to label the protein target cyclophilin A (Fig. 16b).^116^ Without having identified the modified residue, they demonstrated successful labeling with biotin *via* the transfer of tetrazole to the nucleophilic residue (99). In general, the 5-sulfonyl tetrazole moiety exhibited high reactivity towards thiols, suggesting that cysteine residues were most likely modified.^116^

**Fig. 16:**
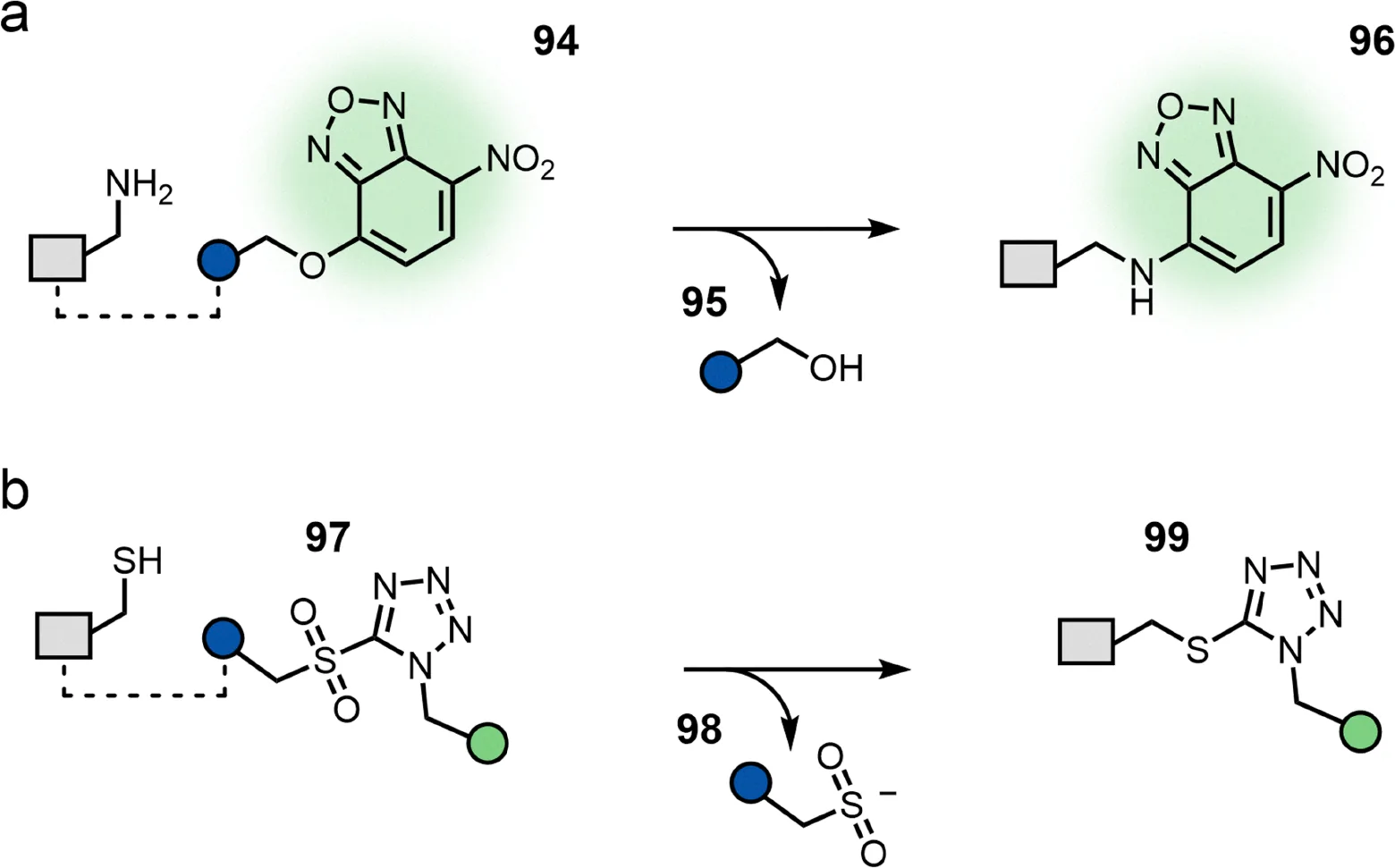
Nucleophilic aromatic substitution *via* (a) a nitrobenzoxadiazole (94) resulting in a “turn-on” fluorescent probe. The nitrobenzoxadiazole is shown explicitly for better illustration of the chemistry, or (b) a sulfonyl tetrazole (97).

#### 3.1.6 Conjugate addition

The design of a “turn-on” fluorescent probe *via* a conjugate addition and lactonization was investigated by Kojima and coworkers (Fig. 17).^117^ Nucleophilic target residues were found to react with the ynoate (100), followed by the hydroxyl and ester group reacting *via* lactonization (101) to form 7-methoxycoumarin, which exhibits increased fluorescence (103, Fig. 17). The reported probe showed reactivity towards cysteine, lysine, histidine, tyrosine and tryptophan. A vitamin D receptor and peroxisome proliferator-activator receptor γ were used as proof-of-concept. Optimization of their probes revealed that substitution with electron-donating groups enhanced fluorescence intensity and shifted the fluorescence band to a longer wavelength.

**Fig. 17:**
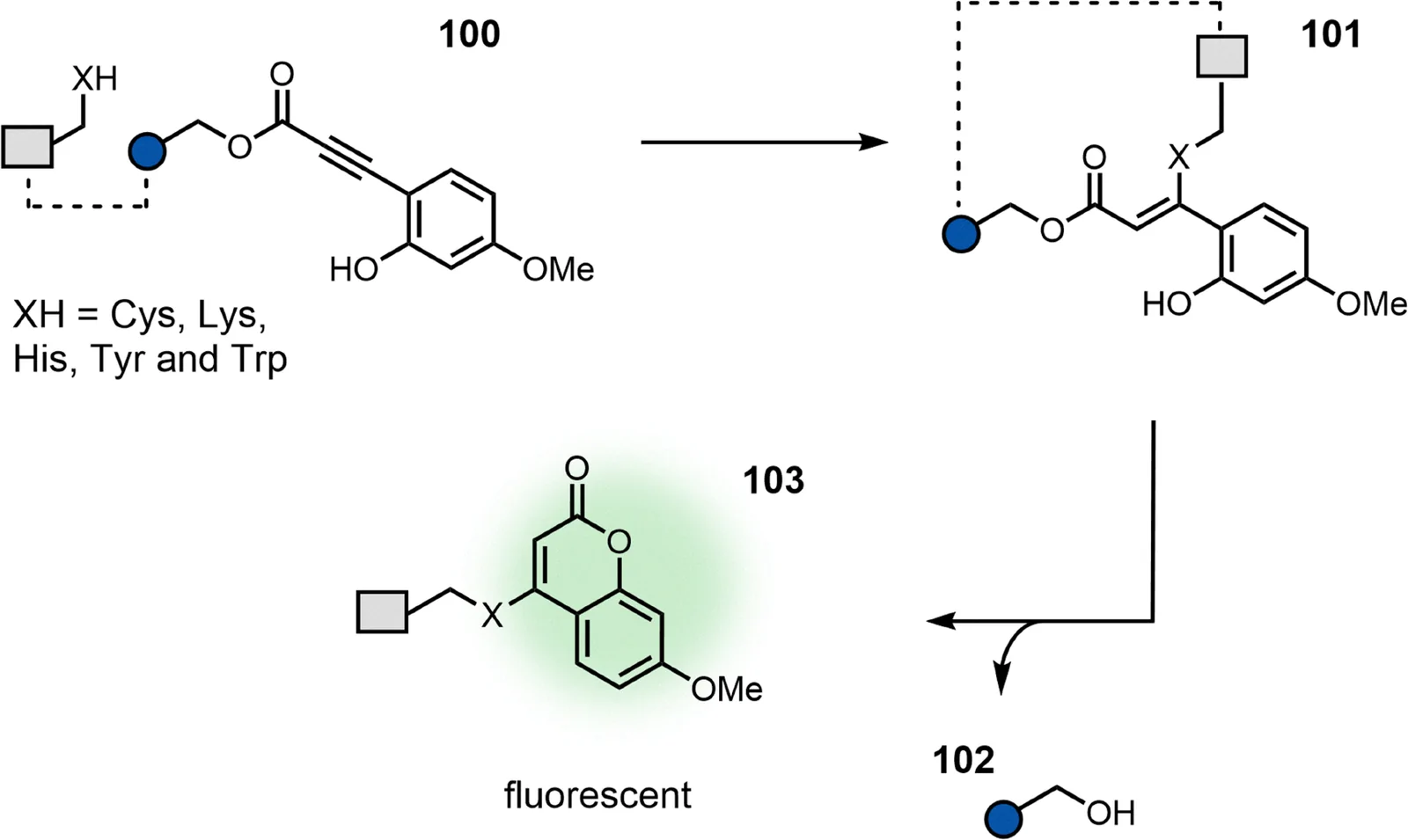
Conjugate addition to a nucleophilic amino acid with an ynoate (100).

### 3.2 Copper-assisted diazo transfer

Proximity-induced transfer was also realized using copper-catalyzed diazo reactions. In contrast to direct transfer reactions, an additional reagent, here Cu(ii), is required to enable chemical modification of a lysine residue on the target protein. This, copper-assisted diazo transfer was applied for the formation of azides on a protein surface using a probe containing imidazole-1-sulfonyl azide (104).^118^ In presence of Cu(ii), this probe specifically reacted with lysines on the protein surface to convert their primary amine into an azide (105, Fig. 18). This allowed subsequent modification (109) of streptavidin, avidin and membrane protein BioY by performing Cu(i)-mediated click reactions with alkynes (107 and 108). Here biotin-transfer was used to test the reaction in protein mixtures, cell lysates and on living cells.^118^

**Fig. 18:**
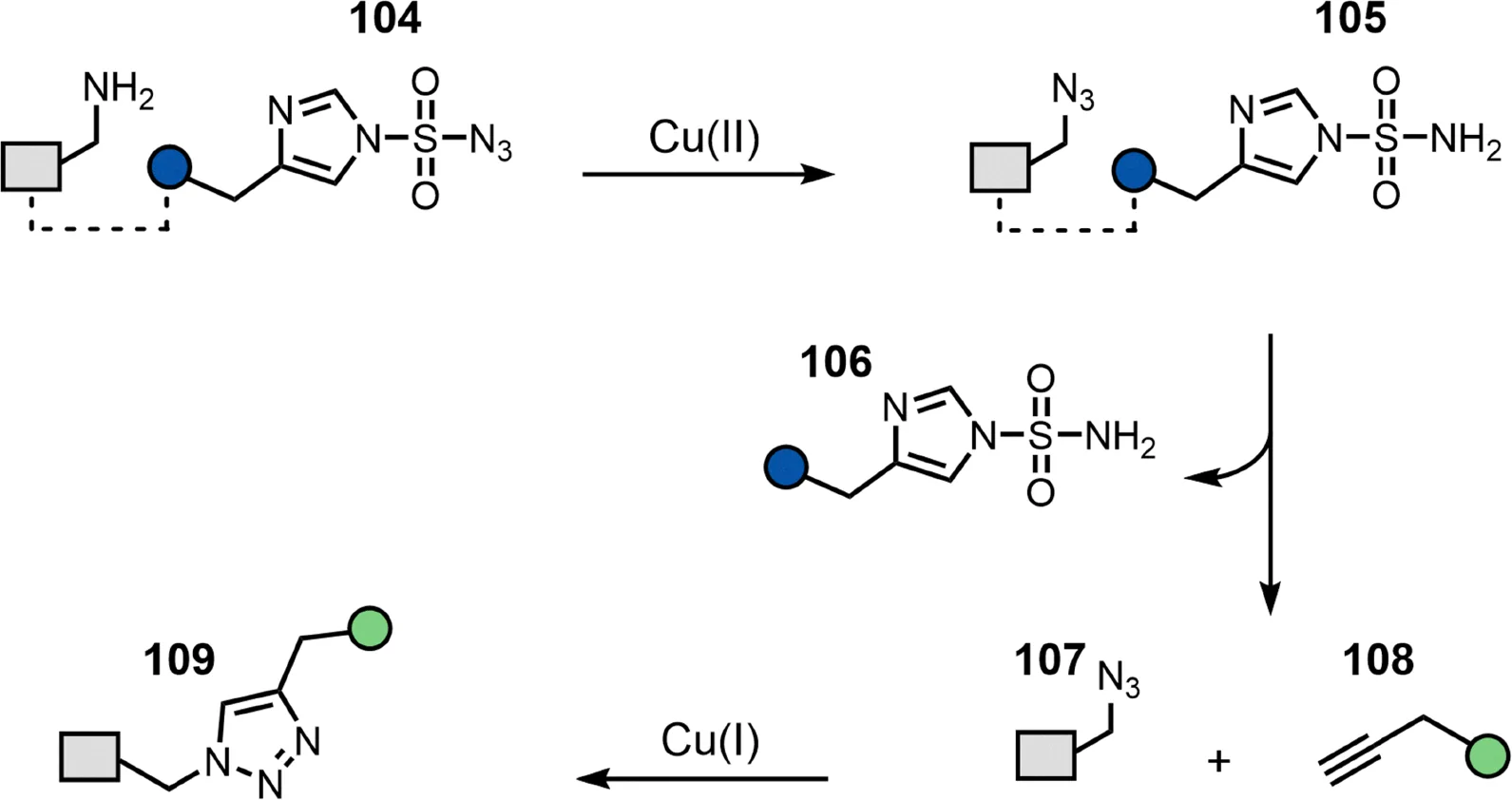
Copper-assisted diazo transfer *via* an imidazole-1-sulfonyl azide (104) providing azide (107) suitable for subsequent functionalization *via* Cu(i)-mediated click reactions.

### 3.3 Photo-induced release and transfer

In the search for alternative methods of selective protein modifications, photochemistry has proven useful when combined with ligand-directed protein modification. In analogy to previously described transfer strategies, the ligand includes a functional moiety and first binds to the target protein. But then, upon light activation, the functional moiety dissociates from the ligand while harboring an *in situ* generated reactive electrophile. This facilitates the subsequent reaction with a nucleophilic protein residue in proximity to the ligand binding site. Notably, the ligand is no longer tethered to the functional moiety, and can thus dissociate from the protein, resulting in a traceless transfer reaction (Fig. 19).

**Fig. 19:**
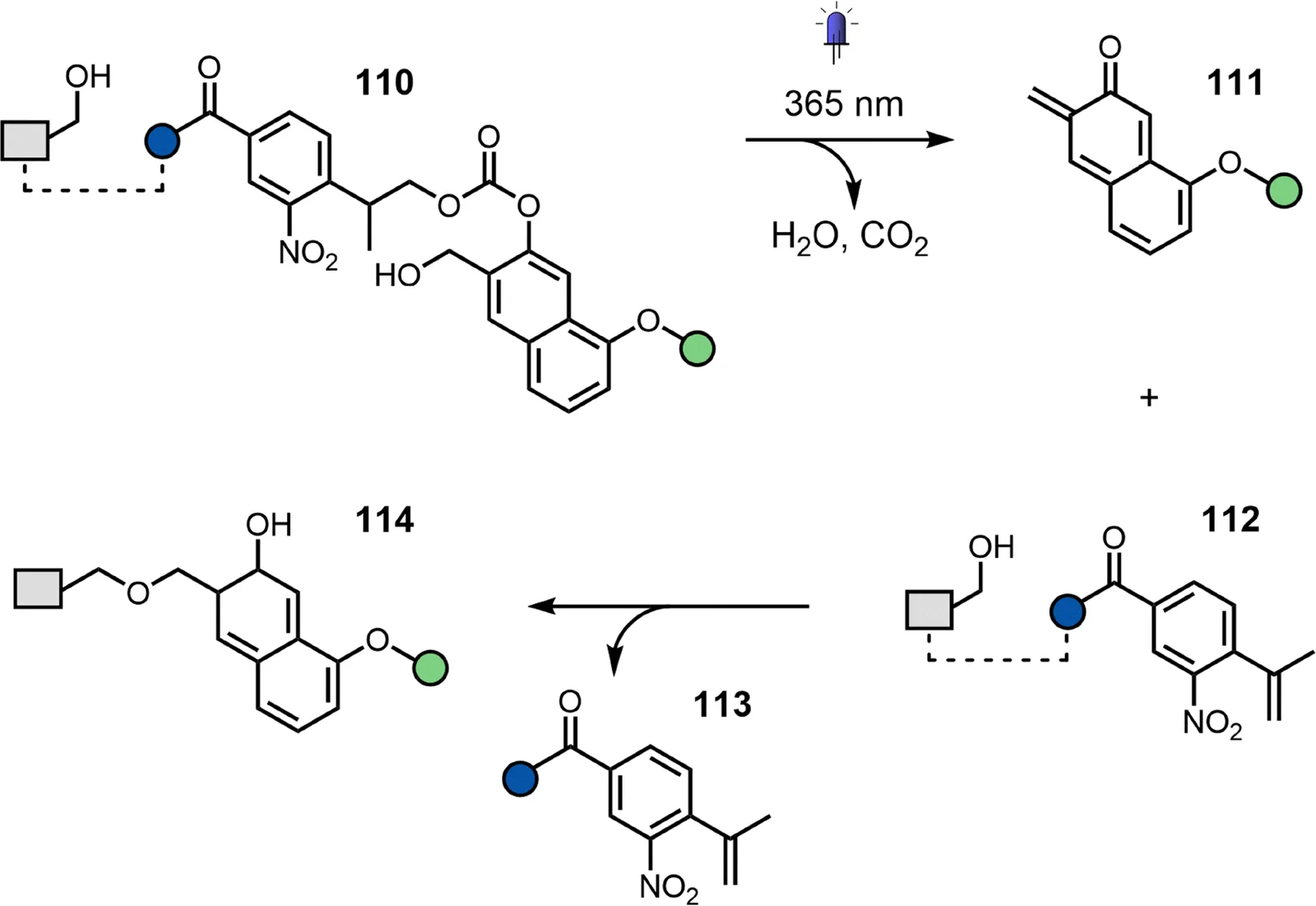
Photo-induced release of *ortho*-naphthoquinone methide (110), followed by nucleophilic attack of a serine residue to result in transfer of the naphthoquinone moiety onto the protein (114).

Photo-induced release and transfer protein labeling was demonstrated by Yu and colleagues.^119,120^ The benzenesulfonamide ligand was designed to contain 2-nitrophenylpropyloxycarbonyl as a photocleavable group,^121^ conjugated with 3-hydroxymethyl-2-napthol (110) bearing the ligand (Fig. 19). Upon irradiation of light at 365 nm, *ortho*-naphthoquinone methide was released (111). The naphthoquinone methide moiety then reacted with a nucleophilic residue in CA, thereby transferring the functional moiety (114). In the absence of UV irradiation, no modification was observed. Peptide mapping analysis showed modification of a single serine residue in proximity of the binding pocket of CA. Various probes such as azides for subsequent click chemistry, fluorophores, and biotin were successfully transferred onto the protein, both on the cell surfaces and in live cells.^119,120^

## 4. Proximity-guided catalysis

In the former examples, proteins were brought together with reaction partners towed by their attachment to ligands, resulting in selective modification of residues in the vicinity of the binding region. Alternatively, it is possible to direct reactions with untethered reaction partners if the ligand instead bears a catalyst, which then enables localized catalytic mechanisms. In this strategy, the ligand-tethered catalyst either recruits or activates an unbound substrate molecule, triggering reactions in the vicinity of the binding site (Fig. 20, top and middle) or it directly activates residues on the protein surface (Fig. 20, bottom). The ligand-tethered catalyst can then dissociate and bind to other target proteins promoting turnover. Selectivity of the reaction is enabled by the proximity of all three reaction partners: the target protein residue(s), the substrate, and the catalyst.

**Fig. 20:**
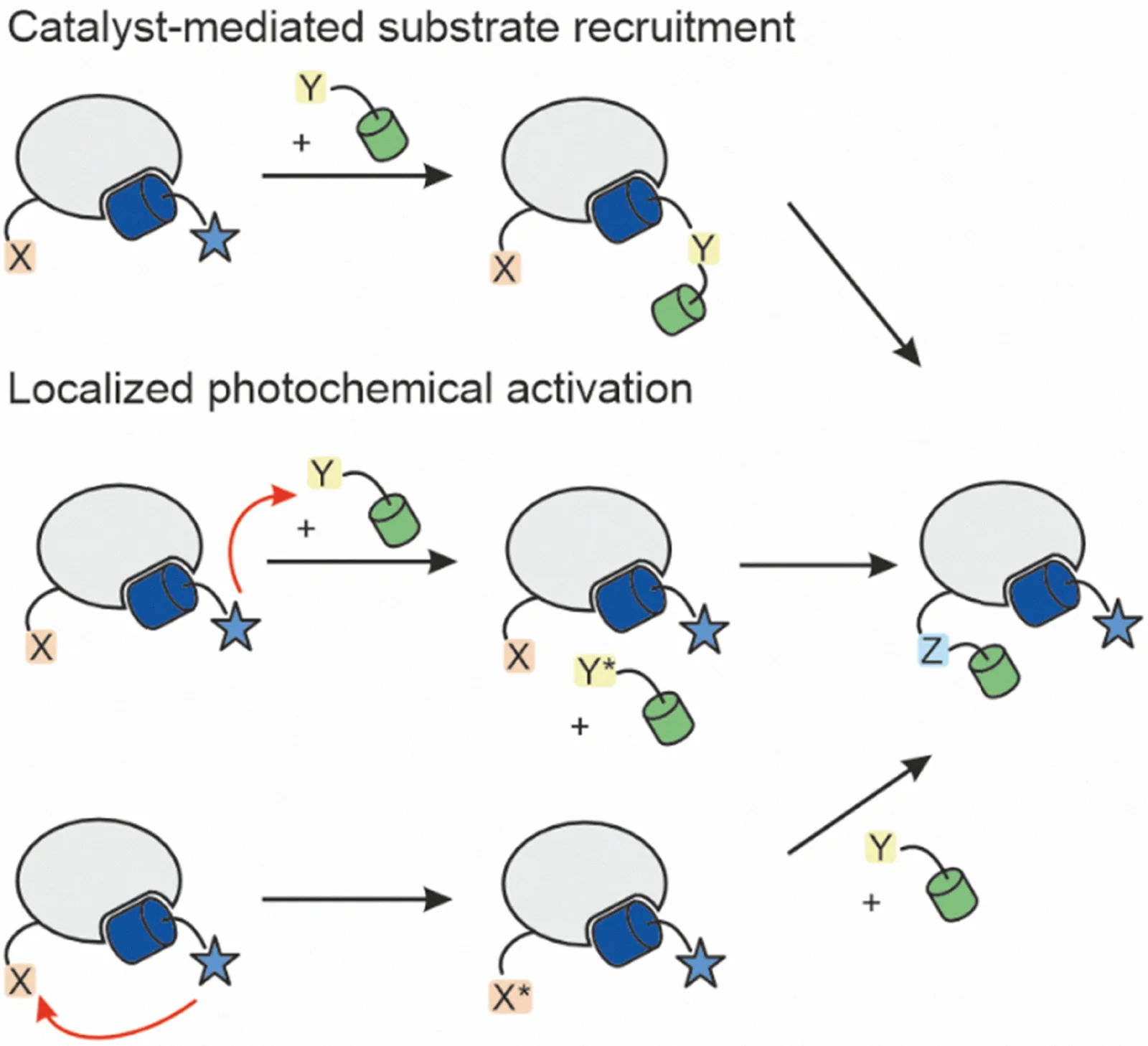
Proximity-guided catalysis can proceed *via* catalyst-mediated substrate recruitment (top), localized photochemical activation (middle) or the direct activation of protein residues by the catalyst (bottom).

### 4.1 Catalyst-mediated substrate recruitment

A straight-forward approach to apply catalysis to ligand-directed protein modification is the use of a catalytic group that forms a covalent intermediate with the substrate, thereby bringing it into proximity of the protein while activating it toward further attack. Usually, the intermediate possesses an electrophilic moiety which can be attacked by nucleophilic residues in the vicinity. These catalysts thus mediate traceless transfer reactions, often acyl transfer, in logical progression from the complementary transfer strategy developed by Hamachi and coworkers (see Section 3 above). The catalytic strategy may be employed to improve reaction rates in cases where the desired transfer modification substrate is insufficiently reactive under the given conditions, without sacrificing selectivity. Furthermore, by decoupling the affinity ligand from the substrate, the catalytic strategy also broadens the viable range of ligands by preventing undesired intramolecular reactions between the two, *i.e.* attack on the electrophilic acyl transfer substrate by a nucleophilic group on the ligand rather than one from the target protein.^13^ Multiple combinations of substrate and catalytic moiety have been reported and are summarized below.

#### 4.1.1 DMAP catalysis

The Hamachi group established this approach to label an array of lectins using DMAP (4-dimethylaminopyridine)-containing catalysts tethered to the relevant saccharide ligands (Fig. 21a).^122^ While held by the tethered ligand in close proximity to the protein binding pocket (115), the DMAP catalyst activates a nearby thiophenyl ester substrate (116) giving an activated intermediate (117), which then transfers its acyl moiety to a nucleophilic residue (119, *e.g.*, the OH group of tyrosine). Subsequent release of the DMAP-saccharide ligand (118) restores the protein's original binding ability. Additionally, ligand multivalency was used to increase binding affinity to the lectin target.^123^ The ligand bearing multiple tethered saccharide units improved coordination to the lectin target and therefore led to increased labeling efficiency of congerins, even when conducted in animal tissue lysate.^122^

**Fig. 21:**
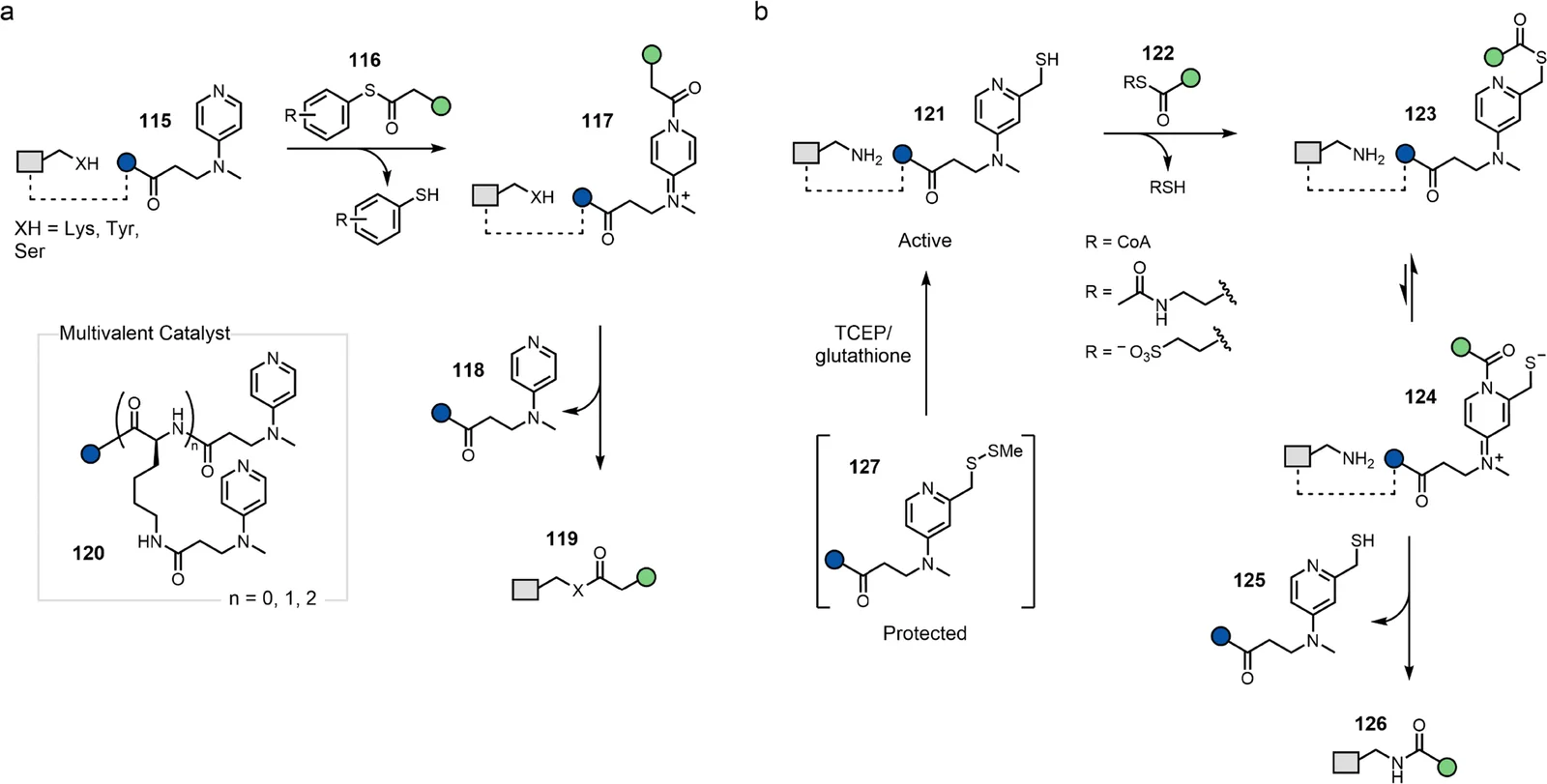
DMAP catalysis. (a) Nucleophilic activation of a thiophenyl ester substrate (116) by a DMAP catalyst (115). Inset: Multivalent DMAP-containing catalysts (120). (b) Nucleophilic activation of a thioester substrate (122) by a mercaptomethyl-modified DMAP catalyst (121).

Furthermore, multivalency of the catalyst was also introduced by incorporating up to three DMAP moieties into one ligand-bound catalyst structure (120, Fig. 21a), thereby increasing the rate of reaction considerably.^124^ Because the number of labeling events on one protein did not increase, it was hypothesized that the tethered DMAP groups might assist each other with deprotonation steps of the reaction, rather than operating independently. With this method, *in vitro* modification was performed for not only congerin II with its lactose ligand, but also SH2 domain with its phosphotyrosine-containing peptide ligand, and FKBP12 with an analog of its immunosuppressant drug molecule ligand FK506 (tacrolimus). Using the ligand-tethered tri-DMAP catalyst, it was further possible to label bradykinin B_2_ receptor and FKBP12 in living cells.^124,125^

Additionally, the group of Kanai demonstrated lysine-selective acylation reactions facilitated by a modified DMAP catalyst bearing a mercaptomethyl group at the 2-position (121, Fig. 21b).^126–128^ Compared to DMAP alone, the added thiol moiety supports activation *via* facile thiol-thioester exchange (123), after which the acyl group is passed to the DMAP pyridine functionality (124). Protection of the free thiol as a methyl disulfide (127) is necessary to prevent dimerization, and the active catalyst can be recovered upon reduction with TCEP or endogenous glutathione. Using DMAP-SH catalyst tethered to a peptide ligand binding to histone H2A-H2B dimer acidic patch region, modification was demonstrated of a single lysine residue (126) on H2B in purified nuclei samples and living cells.^126,128^ Lysine-specific modifications in living cells were further demonstrated on both eDHFR and endogenous heat shock protein 90, by switching the tethered small molecule ligands trimethoprim and PU-H71, respectively.^127^

#### 4.1.2 Pyridinium oxime catalysis

The previous catalyst designs suffered an inherent flaw in that at neutral pH, DMAP exists largely in its protonated, and thus deactivated, form. To address this limitation, the Hamachi group introduced pyridinium oxime as a catalytic group exhibiting a more favorable p*K*_a_ and therefore sufficient nucleophilicity as the deprotonated oximate under relevant conditions (128, Fig. 22).^129^ They also employed NASA acyl donors (129) for the first time in a ligand-directed context, to achieve far more efficient, selective, and biologically relevant reaction conditions.^130^*In vitro* labeling was demonstrated on FKBP12 and CA II, with the pyridinium oxime catalyst tethered to their small molecule ligands.^129^ Again, catalyst multivalency was used to increase the reaction rate (134, Fig. 22), and with optimized conditions, labeling of folate receptor and membrane-bound CA XII was performed in live cells. Lastly, fluorescent labeling of AMPA-type glutamate receptors was successfully achieved in mouse hippocampal and cerebellar tissue slices. External to the scope of this review, pyridinium oxime catalysis has recently been employed for affibody- and antibody-guided protein modification, including additional recognition elements (His-tag) to recruit acyl donor molecules to the site of reaction.^131,132^

**Fig. 22:**
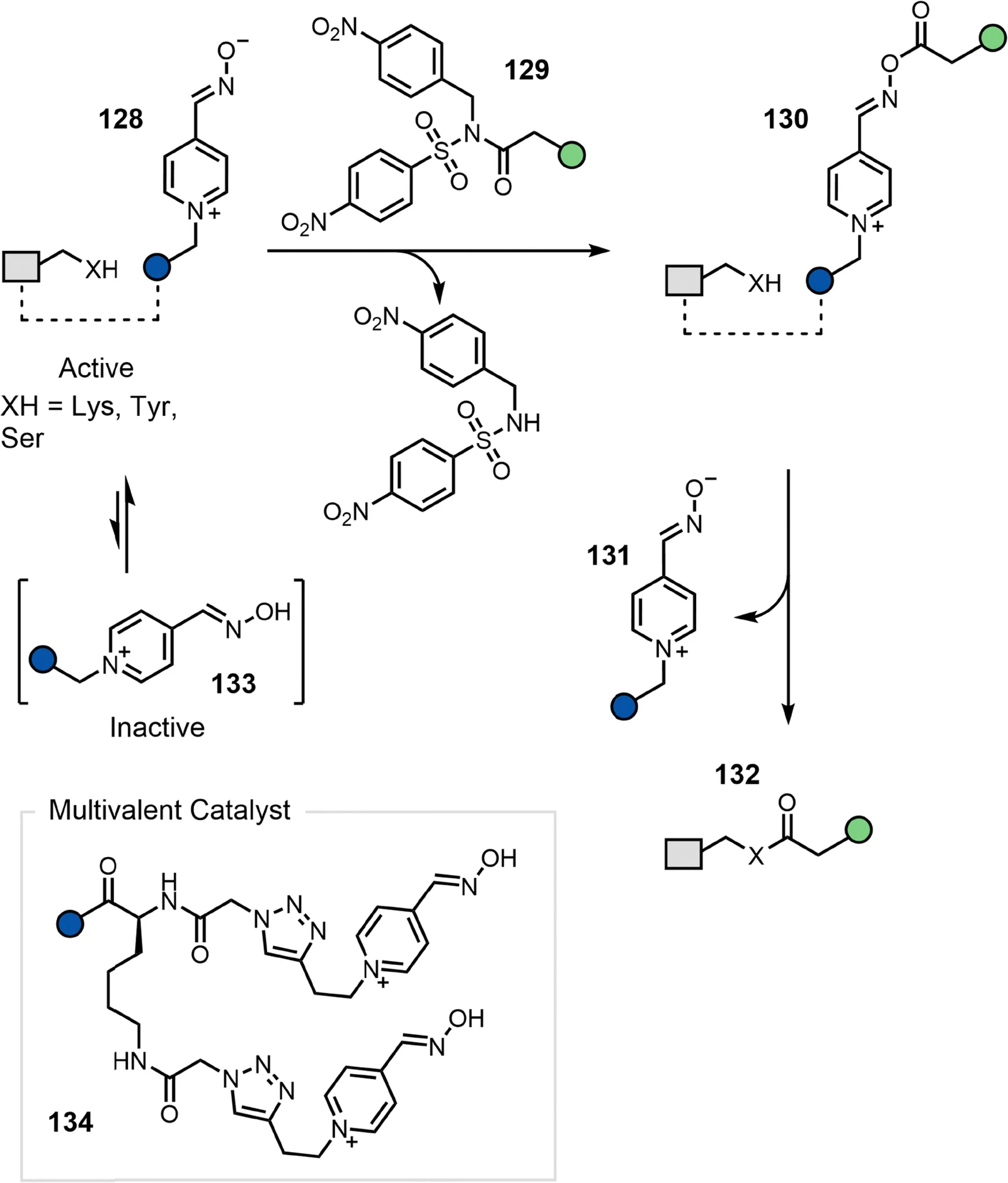
Nucleophilic activation of a NASA acyl donor substrate (129) by a pyridinium oxime catalyst (128). Inset: Multivalent pyridinium oxime-containing catalyst (134).

#### 4.1.3 Hydroxamic acid catalysis

In addition, hydroxamic acid (*N*-hydroxy amide) has been introduced as a versatile nucleophilic catalyst (135) that exhibits increased stability and activity in the intracellular environment (Fig. 23a). Like the pyridinium oxime catalysts described above, the *N*-hydroxyl moiety of hydroxamic acid can be deprotonated in neutral media to access the nucleophilic hydroxamate. The p*K*_a_ is further lowered and tuned by phenyl substitution on the nitrogen, and additional operational groups can be conjugated on the carbonyl side. In the original design of the Kanai group, a conjugated piperidine or pyrrolidine ring reveals the nucleophilic hydroxamate by formation of a putative zwitterionic species, as demonstrated by trimethoprim-ligand-directed lysine modification of eDHFR in cell lysate.^133^

**Fig. 23:**
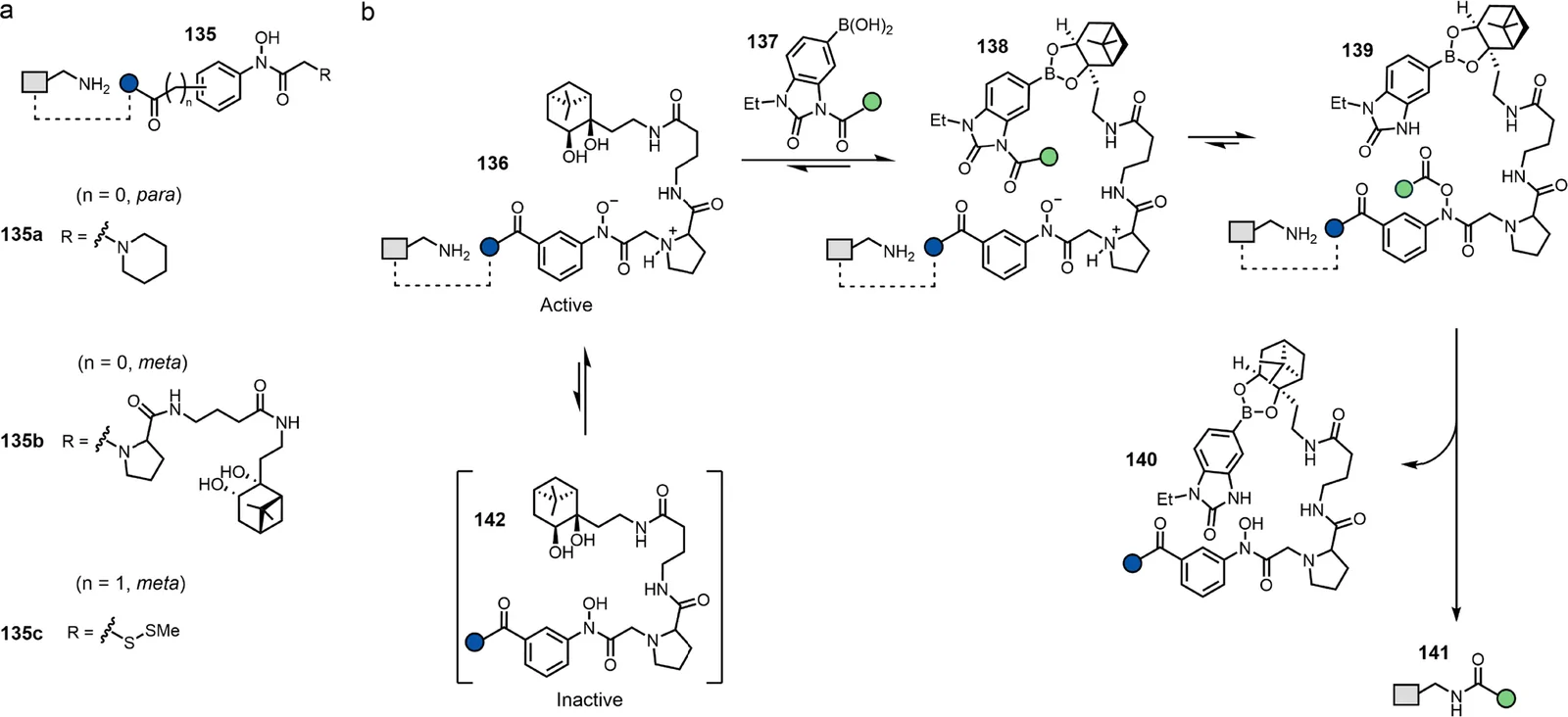
Hydroxamic acid catalysis. (a) Design of hydroxamic acid-containing catalysts (135) with 135a: piperidine for self-activation of the hydroxamate, 135b: pyrrolidine and nopoldiol for recruitment of boronic acid-containing substrates, or 135c: protected thiol for activation of thioester substrates. (b) After boronate-assisted capture (138), nucleophilic activation of an *N*-acyl urea substrate (137) by a hydroxamic acid catalyst (136).

Moreover, selective modification of eDHFR in live cells was achieved by appending an additional diol group to the catalyst structure (136), which reversibly forms a boronate ester (138) with boronic acid-containing acyl donor substrates (137, Fig. 23b).^134^ With this additional substrate-recruiting functionality, mere micromolar substrate concentrations were required for highly efficient modification of a single lysine residue (141). Switching to LANA peptide ligand enabled histone modification instead, with additional groups conjugated for convenient application such as PEG for stability, CPP for cell-permeability, or ligand multivalency for regioselectivity.^135,136^ Note that similar molecular design has also been used to perform ligand-directed ligation reactions.^44^ Furthermore, the specific affinity interaction of eDHFR and trimethoprim/methotrexate-bound catalyst has been recently applied for modification reactions directed by ligand-inserted eDHFR fusion proteins, where both boronate^137^ and thioester^138^ recruitment strategies of donors were demonstrated.

#### 4.1.4 Metal carbene catalysis

In a departure from acylation chemistry, a transition metal-carbene-based strategy has been developed. Here, transition metal catalysts have been used to activate vinyl diazo substrates, forming a reactive metallocarbene intermediate. After early reports demonstrated the ability of rhodium carbenes to modify tryptophan,^139,140^ the versatility of this strategy was elaborated by the group of Ball to include additional residues when performed under affinity modification conditions on polypeptides.^141^ In contrast to residue-specific modification strategies, undiscriminating carbene chemistry is here moderated by the well-behaved dirhodium catalysis and prompted by proximity. The metal catalysts were incorporated into coiled peptide ligands, where carboxylate-containing residues provided one or two of the bridging ligands on a dirhodium(ii,ii) tetracarboxylate paddlewheel structure (Fig. 24). With the metallopeptide ligand in place (143), a vinyl diazo substrate molecule (144) can be activated *via* its complexation to the dirhodium core at one of the axial positions, followed by nitrogen extrusion to form the active metal carbene species (145). Carbene insertion on a nearby residue then occurs to deliver the functional moiety (147). After investigatory studies modifying polypeptides^141,142^ and tagged recombinant proteins,^143^ the desired modification was finally achieved on a series of Src-family SH3 domains.^144,145^ For each SH3 domain, single-site modification was achieved at a tryptophan residue near the peptide binding pocket. Modification could be switched to either of two histidine residues, instead, by adjusting the position of the rhodium catalyst along the peptide ligand. In another example, transcription factor STAT3 was selectively modified at a specific phenylalanine residue located far from the expected drug molecule binding site, suggesting an allosteric inhibition mode.^146^

**Fig. 24:**
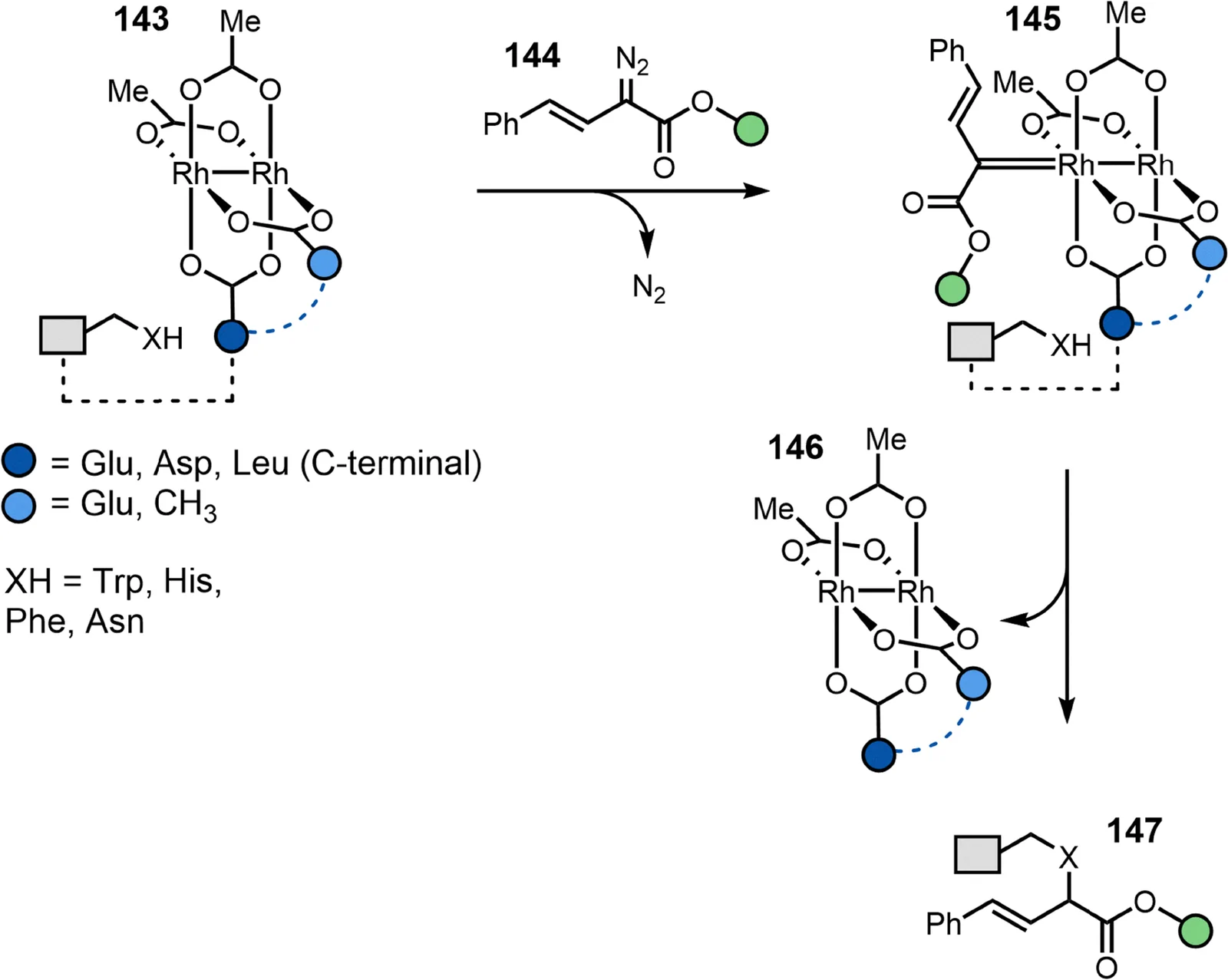
Metal carbene chemistry from a dirhodium catalyst (143) activates a vinyl diazo substrate (144) for addition to diverse residues.

The Ball group also reported an Fc-binding metallopeptide carrying three dirhodium catalysts for selective modification of antibodies.^147^ A range of IgG antibodies were labeled, each at a single asparagine residue on the Fc fragment. Similar to the multivalent DMAP catalysts above (Section 4.1.1), the three rhodium catalysts were necessary for an efficient reaction, but did not appear to operate independently or cause multiple labeling events. It should be noted that biotin interfered with the dirhodium carbene intermediate in certain metallopeptide ligands, causing nonspecific labeling when direct biotinylation was attempted.^145^ Installation of an alkyne tag, instead, provided a click handle for CuAAC reactions with desired functionalities, such as component parts for antibody-drug conjugates (ADC). Despite their remarkable versatility, dirhodium-based modification strategies remain susceptible to reaction with thiol-containing biomolecules. Cysteine residues have been reported to promote decomposition of the dirhodium core and formation of rhodium–thiolate species, potentially limiting catalyst performance in complex biological environments.^148^

### 4.2 Localized photochemical activation

In an alternative approach, the catalytic moiety is not used to bind the substrate, but to locally generate highly energetic intermediate species which quickly react with suitable nearby protein residues. Relevant short-lived reactive intermediates may include species such as radicals, carbenes, nitrenes, or singlet oxygen. Depending on the effective diffusion radius of the reactive species, labeling may be confined to the ligand binding site, or it may be extended to label nearby interacting species in the media.^149^ Therefore, applications of this strategy often lie in fields of study outside the scope of this review under the umbrella of proximity labeling, such as mapping of protein-protein interactions. However, the catalytic mechanisms will be here summarized as they represent important strategic contributions to the field, and examples will be highlighted that focus on targeted modification of a protein of interest rather than its interactome. Catalytic activity can also be applied directly to the target protein itself, in which case reactive modes are limited by the chemical nature of the conveniently-positioned protein residues. Methods involving the incorporation of unnatural or pre-determined residues into the protein are outside the scope of this review.

In this context, photocatalysts offer uniquely suitable modes of reactivity, and these may be divided into energy transfer and electron transfer types, both of which have been applied for protein modification.^150^ Depending on the energy levels of the interacting species, however, they may co-occur. It may therefore be difficult to untangle all possible mechanisms for an observed transformation, especially in complex biological media containing water, molecular oxygen, and other endogenous interactors. Energy transfer catalysis, or photosensitization, makes use of the short-range transfer of triplet energy from the excited-state photocatalyst to generate activated intermediates.^151,152^ Various transition metal and organic photocatalysts are capable of absorbing visible light and converting to long-lived triplet excited states *via* efficient intersystem crossing, or, in some cases, absorbing in the NIR range to directly populate the triplet state *via* spin-forbidden excitation.^153,154^ Reactivity is commenced by transfer of the triplet energy (*via* Dexter exchange) to a susceptible target, such as a carbene precursor or molecular oxygen. In contrast, electron transfer catalysis, or photoredox, employs single-electron transfer (SET) mechanisms to generate free radicals.^155,156^ In this case, photocatalysts, selected according to their pertinent redox potentials, are excited by visible light, increasing both the oxidation and reduction capabilities relative to their ground state. The excited-state photocatalyst then enters reductive or oxidative quenching cycles, respectively, to form activated radical intermediates.

#### 4.2.1 Carbene generation *via* energy transfer

Rather than metallocarbene intermediates described in Section 4.1.4, an alternative strategy is to release free carbenes from the carbene precursor substrate, which react quickly at residues within their immediate vicinity (Fig. 25). Carbenes diffusing beyond their effective labeling radius^157^ will be quickly consumed by their O–H insertion reaction with water. Thus, specificity of the reaction is achieved *via* the transient availability of an otherwise unspecific reactant. Although in this approach the specific residues modified are not always disclosed, elucidation of the labeled residues for mapping of the actual binding site region may be accomplished by employing appropriate digestion and mass spectrometry methods.

**Fig. 25:**
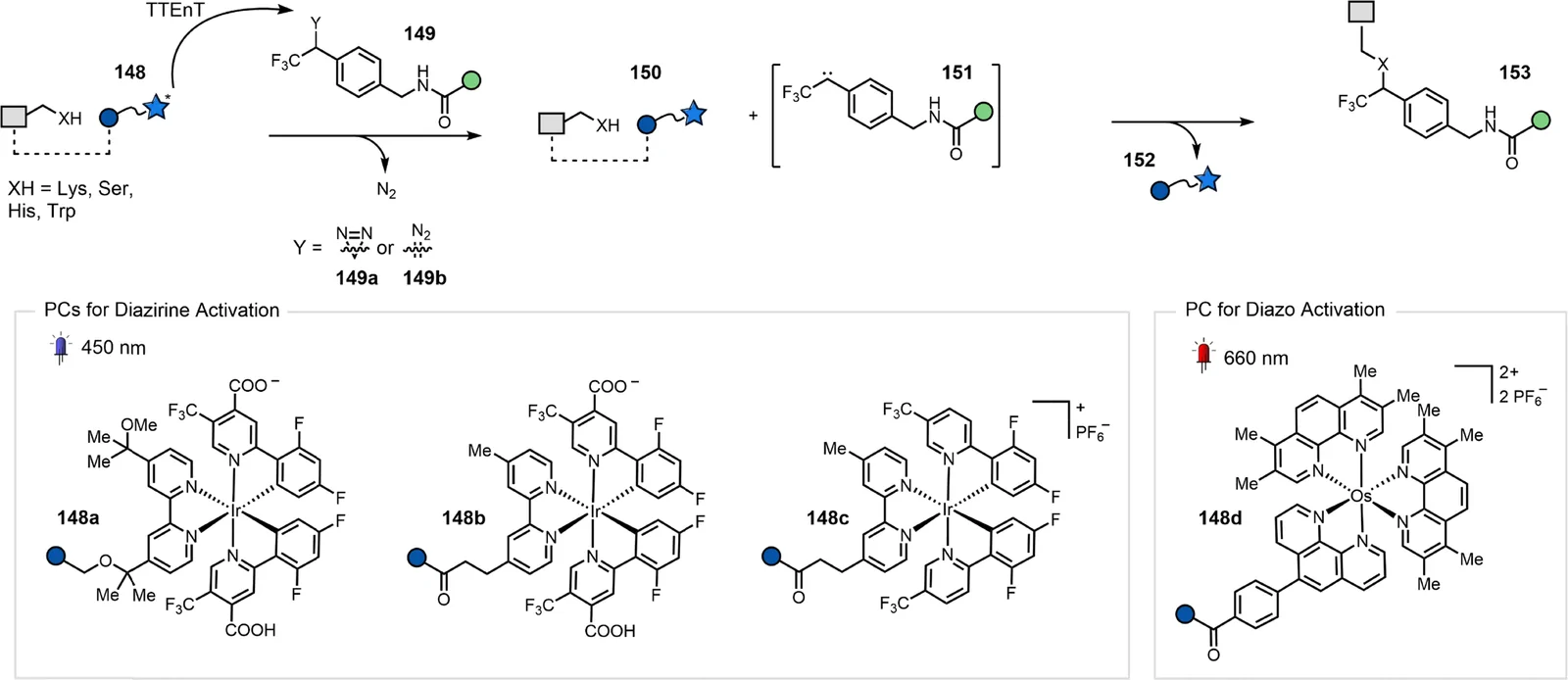
Photocatalytic generation of carbenes derived from aryl(trifluoromethyl) diazirine (149a) and diazo (149b) substrates for addition to diverse residues. Inset: Blue-light-activated catalysts for diazirine activation (148a–c) and red-light-activated catalyst for diazo activation (148d).

After the MacMillan group reported iridium-sensitized activation of diazirines for antibody-guided labeling,^158^ this strategy was applied in combination with small molecule ligands both *in vitro* and in live cells.^14,159^ In these reports, blue light irradiation excites the iridium photocatalyst (148a–c), which subsequently transfers triplet-state energy to a nearby aryl(trifluoromethyl)diazirine substrate (149a) *via* short-range Dexter energy transfer, initiating nitrogen extrusion and carbene release (151). The degree of labeling is linearly correlated to irradiation time, due to the substrate stability in the absence of photosensitization. Using this strategy, MacMillan and coworkers demonstrated selective labeling and binding-site mapping of bromodomain-containing protein 4 with inhibitor-tethered iridium catalyst. Similarly, an array of small molecule ligands was used for targeted modification of kinases, tubulins, membrane proteins, molecular glue targets, and STAT3 at its allosteric binding site. More recently, energy-transfer-based photoproximity labeling strategies using compact organic photocatalysts have enabled the activation of diazirines to generate carbenes.^160^ These systems provide a biocompatible alternative to metal-based photocatalysts for proximity labeling in live cells.

In place of diazirines, diazo carbene precursor substrates (149b) may also be used. In this case, the more reactive diazo moiety requires a lower triplet energy sensitization, and therefore a red-light-activated osmium complex (148d) is more suitable for its selective activation. In recent work from Rovis and coworkers, at least 11 amino acids were amenable to insertion of carbenes derived from aryl(trifluoromethyl)diazo substrates.^161^ Selective modification of CA was achieved in cell lysate using this method, with the goal of widely-applicable directed protein modification under cell-penetrating red light.

#### 4.2.2 Singlet oxygen generation *via* energy transfer

When triplet energy transfer occurs with ground-state triplet oxygen, reactive singlet oxygen is generated, which can efficiently oxidize biomolecules. Also referred to as Type II photooxidation, singlet oxygen reactivity on proteins is implicated in biological systems naturally and, in the case of photodynamic therapy (PDT), remedially.^162,163^ The oxidized residues are susceptible to further reactivity from probe-bearing substrate molecules (Fig. 26). In proximity labeling systems, the singlet oxygen is generated and restricted to the area immediately around the catalyst due to its limited lifetime and diffusion radius.^152^

**Fig. 26:**
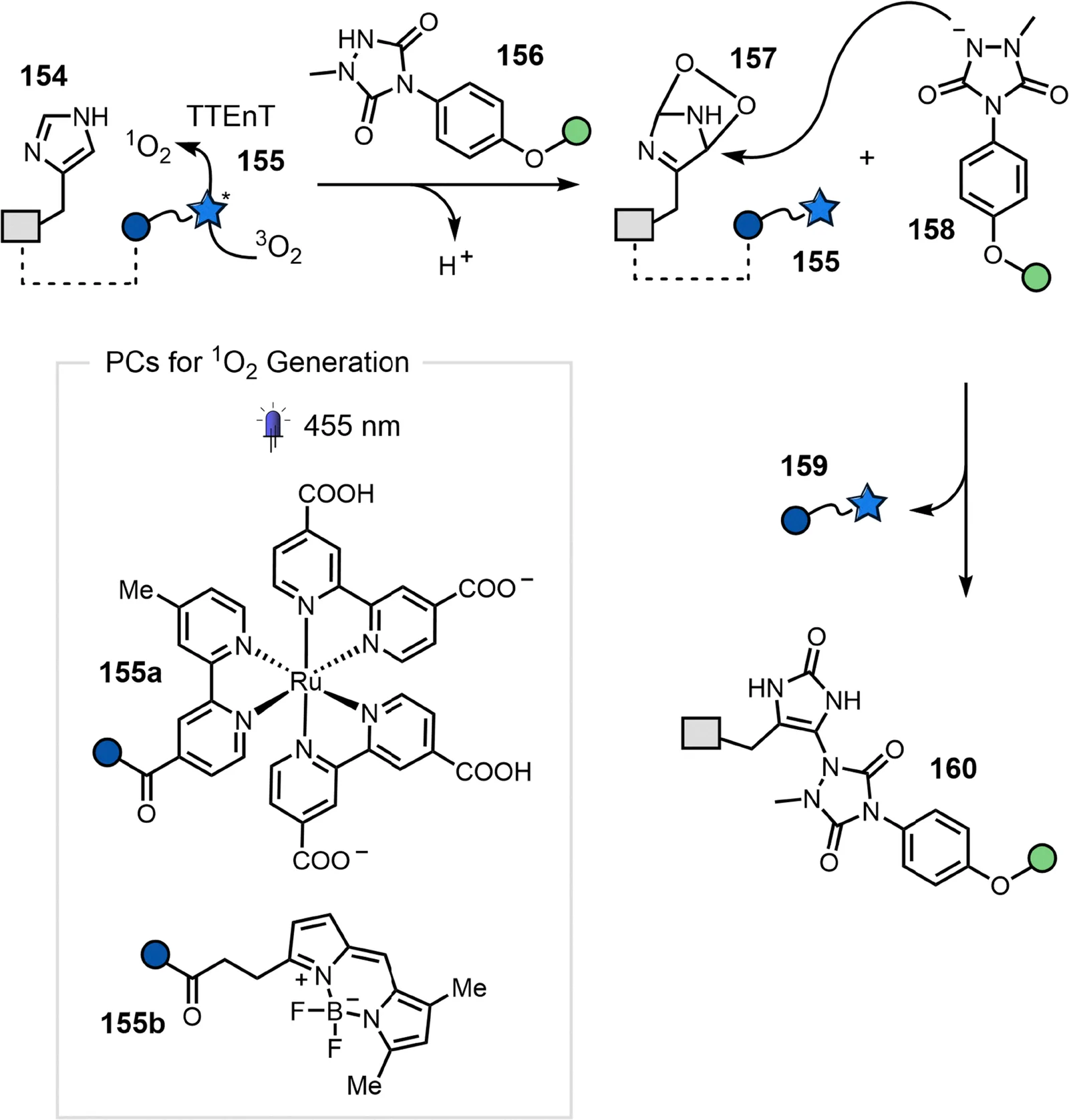
Photocatalytic generation of singlet oxygen for addition of a 1-methyl-4-arylurazole substrate (156) to oxidized histidine residues (157). Inset: Blue-light-activated catalysts for generation of singlet oxygen (155a–b).

Recently, Nakamura and Sato groups reported targeted modification of histidine residues using catalytically generated singlet oxygen.^164,165^ Upon light irradiation, singlet oxygen is produced by triplet energy transfer from a ligand-tethered photosensitizer (155) such as a ruthenium complex (155a) or an organic dye like BODIPY (155b). Upon cycloaddition to the imidazole ring of a nearby histidine residue, the resulting endoperoxide (157) becomes activated toward nucleophilic attack by a substrate (158) in Umpolung fashion. With this strategy, blue-light-activated ruthenium or BODIPY photocatalysts facilitated modification of Trastuzumab's Fc domain at three histidine residues (160) close to the binding site of the Fc-binding ligands. In the case of histidine modification with ruthenium photocatalyst, an additional design element was employed, wherein the photocatalyst and ligand were immobilized side-by-side on magnetic beads for ease of purification.^164^ Although the authors differentiate this method from “ligand-directed” labeling, it might be thought of as such since the origin of specificity of the reaction is still attributed to the ligand-catalyst connection. In this case, the bead surface might be considered to be the tether.

#### 4.2.3 Radical generation *via* photoredox catalysis

Photoredox catalysis is a versatile tool that may be used to form reactive radical intermediates originating from either the substrate molecule or residues on the protein itself. In the case of substrate activation, reaction control may vary depending on appropriate matching of redox potentials for the chosen substrate-catalyst pair, and spatial precision of the modification may vary depending on the effective diffusion radius of the radicalized substrate. In the case of protein activation, residue(s) susceptible to SET must be located in the vicinity.^150,166^ Often, the identity of the involved radicals is not definitively known, and multiple reaction pathways may be possible. Likewise note that in some cases, energy transfer mechanisms may operate alongside photoredox.^167,168^ If the reaction is not conducted under inert atmosphere, the presence of oxygen in biological media may quench triplet states, diverting redox pathways or causing product mixtures resulting from photooxygenation. Oxygen may also participate in the redox cycle itself by accepting an electron to form superoxide.

The use of ligand-tethered photoredox catalysts was first explored by Nakamura and coworkers for the selective modification of CA at tyrosine, where a benzenesulfonamide ligand was tethered to a photocatalytic ruthenium complex (161, Fig. 27a).^169^ Upon excitation with visible light, the excited-state Ru(ii)* is oxidized to Ru(iii) *via* loss of an electron to a sacrificial oxidant, which can be ammonium persulfate (APS) or molecular oxygen, although less efficiently.^170^ SET to the oxidized catalyst occurs from either a nearby tyrosine residue (163) or the labeling substrate molecule (165), producing a radical intermediate that is quickly trapped (167). An analogous approach was applied by Bon and coworkers to label cysteine (164) in the presence of APS.^171^ With this protocol, the MCL-1 protein was labeled using a photocatalyst tethered to a BH3 domain peptide ligand, identifying a single cysteine residue as the site of modification (168).

**Fig. 27:**
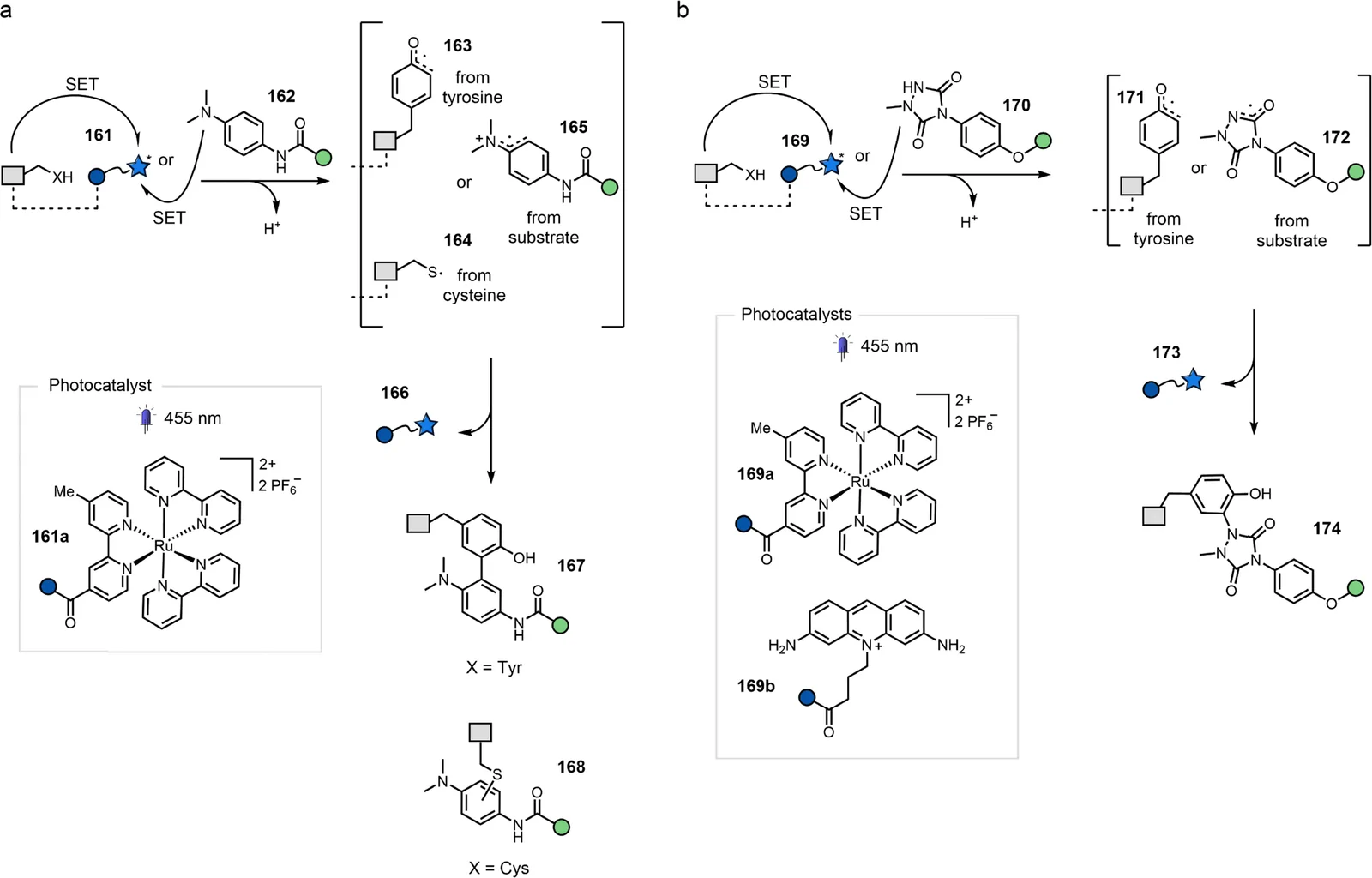
(a) Single-electron oxidation by an excited-state photocatalyst (161) enables radical trapping of a residue (163, 164) or an *N*′-acyl-*N*,*N*-dimethyl-1,4-phenylenediamine substrate (165). Inset: Blue-light-activated catalyst for redox reactions with tyrosine and cysteine (161a). (b) Single-electron oxidation by an excited-state photocatalyst (169) enables radical trapping of a residue (171) or a 1-methyl-4-arylurazole substrate (172). Inset: Blue-light-activated catalysts for redox reactions with tyrosine (169a–b).

In the previous examples, labeling was not always confined to the immediate vicinity of the photocatalyst, an observation that could be explained by the relatively stable nature of the radicals produced by the phenylenediamine substrate. Therefore, Nakamura and coworkers investigated labeling reagents that upon oxidation would form a less stable radical with an accordingly smaller diffusion radius, identifying 1-methyl-4-arylurazole as an optimal structure (170, Fig. 27b).^172^ In this work, CA was selectively modified at the tyrosine residue (174) closest to the ligand binding site. Additionally, a subsequent work identified acriflavine as a selective photocatalyst for SET oxidation,^173^ precluding the competing singlet oxygen pathway with ruthenium.^168^ Although this has not yet been used for ligand-directed modification, it provides an intriguing target for a selective and cell-permeable radical-based proximity labeling platform. It is also notable that the reactions with ruthenium photocatalyst and both phenylenediamine^174^ and urazole^175,176^ substrates were applicable to the convenient method of magnetic bead immobilization.

Lastly, it should be noted that Rovis^177^ and MacMillan^178^ groups have also developed related red-light-activated labeling methods with nitrenes or aminyl radicals unleashed from aryl azide substrates. Here, single-electron reduction of the azide from an excited-state osmium or a reduced tin catalyst initiates nitrogen extrusion and release of the reactive group. However, these methodologies have so far been used for antibody-directed microenvironment mapping studies, where the longer lifetime of nitrenes and aminyl radicals leads to less specific labeling when compared to carbenes (Section 4.2.1).

## 5. Conclusions and outlook

Ligand-directed protein modification has matured into a versatile platform for the site-selective functionalization of native proteins without relying on genetic engineering. A central feature is the use of a binder to position the reactive group near a target residue, increasing their effective molarity and thereby promoting covalent bond formation. The efficiency of covalent modification depends on several factors associated with target recognition and probe orientation, including ligand affinity and binding kinetics, the accessibility of the target residue, and the positioning of the reactive group. In parallel, the intrinsic reactivity and selectivity of the reactive group, as well as the kinetics of its reaction with the target residue, are important determinants of covalent modification. In addition, the probe concentration required for efficient modification, which is influenced, among other factors, by the affinity of the ligand for the target protein, can substantially affect performance, as higher probe concentrations may increase background reactions. Accordingly, the optimal balance between ligand affinity, covalent reaction kinetics, and reactivity depends on the intended application and the complexity of the biological environment. Despite sharing a common reliance on proximity effects, the three principal strategies differ in their design logic, scope, and practical implications.

Ligation reactions represent the most conceptually straightforward approach, in which a ligand positions a reactive group to form a covalent bond with a proximal residue. This strategy benefits from its conceptual simplicity and broad reaction scope, enabling robust modification across diverse protein classes and environments. However, the permanent attachment of the ligand causes a significant steric footprint which can interfere with protein function or downstream applications. While ligation-cleavage sequences partially address this limitation, they typically introduce additional synthetic complexity and require careful control over orthogonal reactivity.

Transfer reactions overcome this limitation by enabling installation of functional moieties with concomitant release of the ligand. This results in minimally perturbed protein conjugates while leveraging the chemical diversity of available electrophiles, thereby increasing flexibility. Several strategies have demonstrated effectiveness in complex biological settings, including live cells. Nevertheless, transfer reactions often require a delicate balance between reactivity and stability: highly reactive systems risk off-target labeling, whereas less reactive systems can suffer from slow kinetics and incomplete conversion. Furthermore, the design of efficient leaving groups and optimized linker architectures remains a challenge that strongly influences performance.

Proximity-guided catalysis introduces an additional conceptual layer by decoupling the ligand from the reactive substrate, employing ligand-tethered catalysts as mediators. This approach expands the accessible chemical space, allowing the use of substrates and ligands that would be incompatible with direct tethering. Recently, catalytic modification systems have advanced to enable reactions in live cells and tissue environments, as well as the use of convenient “turn-on/turn-off” photochemical activation modes to provide spatiotemporal control. At the same time, these systems are inherently more complex, requiring careful optimization of catalyst activity, substrate compatibility, and spatial confinement of diffusive reactive intermediates.

The biological context strongly influences the requirements for ligand-directed protein modification. For the modification of individual purified proteins, selectivity against competing proteins is generally not a concern, allowing relatively reactive electrophiles and catalytic systems to be used for rapid modification. In complex biological environments, reagents must discriminate the target protein from chemically similar sites across the proteome, placing greater demands on ligand positioning, reactivity, and reaction kinetics. Here, the balance between ligand recognition, spatial confinement, electrophile reactivity, and reaction kinetics becomes critical, often resulting in slower modification rates. Ligation reactions offer a conceptually straightforward approach for robust site-selective modification, although the permanent attachment of the ligand can introduce a substantial steric footprint. Transfer reactions can overcome this limitation by releasing the ligand after modification, making them attractive when minimal perturbation of the protein conjugate is desired, although often at the cost of more laborious probe synthesis. Proximity-guided catalysis further expands the accessible reaction space and can be particularly useful when the use of stoichiometric amounts of ligand is undesirable.

Looking ahead, the continued evolution of ligand-directed protein modification will depend increasingly on the ability to achieve predictable performance in complex biological environments. In particular, the field faces a fundamental challenge in balancing reactivity, selectivity, and biocompatibility. Achieving efficient modification under native conditions at low occupancy and in the presence of competing endogenous species therefore remains a central objective. In addition, expanding the scope of addressable amino acid residues will be crucial. Another challenge lies in improving the predictability of modification outcomes. Although ligand binding provides a powerful means of enabling local proximity, successful protein modification is ultimately governed by a complex interplay of binding geometry, residue accessibility, local microenvironment, and reaction kinetics. In this context, insights from the design of proteolysis targeting chimeras (PROTACs), a class of bifunctional ligands that trigger protein degradation, highlight the importance of binding geometry, cooperativity, and spatial organization in translating proximity into efficient and selective chemical reactivity.^179,180^

Further advances will require tighter integration of structural biology, computational modeling, and chemoproteomic analysis to enable a more rational design of ligand-directed reagents and catalysts. In parallel, advances in ligand discovery can be expected to substantially expand the range of addressable protein targets.^181–184^ An important long-term direction will be the development of strategies that combine broad residue scope with high positional precision and minimal perturbation of protein function. Currently, methods often involve trade-offs between residue selectivity, reaction efficiency, and structural footprint, and few approaches are universally applicable across different protein classes. Here, the convergence of ligand-directed chemistry with advances in synthetic biology, proteomics, and therapeutic targeting is likely to transform these methodologies from specialized labeling tools into general platforms for controlling and interrogating protein function directly in native biological systems.

## Conflicts of interest

T. N. G. is a co-founder of Incircular B.V., a university spin-off commercializing INCYPRO, a chemical protein engineering technology developed in his group.

## Data Availability

No primary research results, software or code have been included and no new data were generated or analysed as part of this review.
